# Cytotoxic metabolites from *Sinularia levi* supported by network pharmacology

**DOI:** 10.1371/journal.pone.0294311

**Published:** 2024-02-06

**Authors:** Mingna Sun, Miada F. Abdelwahab, Jianye Zhang, Mamdouh Nabil Samy, Nada M. Mohamed, Islam M. Abdel-Rahman, Faisal Alsenani, Usama Ramadan Abdelmohsen, Basma Khalaf Mahmoud

**Affiliations:** 1 Guangzhou Municipal and Guangdong Provincial Key Laboratory of Molecular Target & Clinical Pharmacology, The NMPA and State Key Laboratory of Respiratory Disease, School of Pharmaceutical Sciences and the Fifth Affiliated Hospital, Guangzhou Medical University, Guangzhou, China; 2 Department of Pharmacognosy, Faculty of Pharmacy, Minia University, Minia, Egypt; 3 Department of Pharmaceutical Chemistry, Modern University for Technology and Information (MTI), Cairo, Egypt; 4 Department of Pharmaceutical Chemistry, Faculty of Pharmacy, Deraya University, New-Minia, Minia, Egypt; 5 Department of Pharmacognosy, College of Pharmacy, Umm Al-Qura University, Makkah, Saudi Arabia; 6 Department of Pharmacognosy, Faculty of Pharmacy, Deraya University, New Minia, Egypt; Ahram Canadian University, EGYPT

## Abstract

The *in-vitro* anti-proliferative evaluation of *Sinularia levi* total extract against three cell lines revealed its potent effect against Caco-2 cell line with IC_50_ 3.3 μg/mL, followed by MCF-7 and HepG-2 with IC_50_ 6.4 μg/mL and 8.5 μg/mL, respectively, in comparison to doxorubicin. Metabolic profiling of *S*. *levi* total extract using liquid chromatography coupled with high-resolution electrospray ionization mass spectrometry (LC-HR-ESI-MS) revealed the presence of phytoconstituents clusters consisting mainly of steroids and terpenoids (1–20), together with five metabolites 21–25, which were additionally isolated and identified through the phytochemical investigation of *S*. *levi* total extract through various chromatographic and spectroscopic techniques. The isolated metabolites included one sesquiterpene, two steroids and two diterpenes, among which compounds prostantherol (21) and 12-hydroperoxylsarcoph-10-ene (25) were reported for the first time in *Sinularia* genus. The cytotoxic potential evaluation of the isolated compounds revealed variable cytotoxic effects against the three tested cell lines. Compound 25 was the most potent with IC_50_ value of 2.13 ± 0.09, 3.54 ± 0.07 and 5.67 ± 0.08 μg/mL against HepG-2, MCF-7 and Caco-2, respectively, followed by gorgosterol (23) and sarcophine (24). Additionally, network analysis showed that cyclin-dependent kinase 1 (CDK1) was encountered in the mechanism of action of the three cancer types. Molecular docking analysis revealed that CDK1 inhibition could possibly be the reason for the cytotoxic potential.

## Introduction

The value of marine natural products as drug leads has inherently increased in the past decades [1]. They apparently proffer both chemical diversity and noteworthy biological potential [2]. Marine organisms represent an extraordinary treasure for the discovery of new bioactive compounds with therapeutic and industrial importance [3]. Consequently, myriads of novel and interesting metabolites have been isolated and identified [4]. The marine world is exceptionally rich in bio diverse species available for chemical and biological exploration [5]. Among the unique marine ecosystems, coral reefs which have been regarded as one of the most productive biological communities [6]. Soft corals (also known as sea fans, sea whips or sea plumes) are the most dominant invertebrates usually inhabiting warm seawaters coral reefs or rocks in tropical and subtropical regions [7]. Soft corals develop several mechanisms to resist the fierce conditions involving attack and defence, symbiosis and allelopathy as well as the competition for nutrients and space. They mainly rely on the production of defensive chemicals, released to their surrounding environments which are toxic to some predators or adjacent hard corals. Actually, some of these metabolites may be valuable to humans [8, 9]. These facts brought scientists’ attention to chemically and biologically investigate these interesting marine invertebrates. Subsequently, this has led to the isolation of biologically promising marine natural products with unprecedented chemical structures [10].

The genus *Sinularia* includes a group of soft corals belonging to the phylum Cnidaria, class Anthozoa, subclass Octocorallia, order Alcyonacea and family Alcyoniidae [11, 12]. They are one of the most widespread soft corals in shallow waters from east Africa to western Pacific, rarely found in huge aggregates. This genus comprises approximately 90 species of which more than 50 have been chemically investigated [6, 13]. As reported, secondary metabolites which have been isolated from *Sinularia* are characterized by their intriguing and diverse structural features, including sesquiterpenoids [14, 15], norsesquiterpenoids [16, 17], diterpenoids [18, 19], norditerpenoids [16, 20], steroids [21, 22] and other chemical compounds [23, 24]. Importantly, this metabolic diversity has been demonstrated to display potential biological activities such as cytotoxic [25, 26], anti-inflammatory [27, 28], neuroprotective [29, 30], antifouling [31, 32] and antimicrobial [33, 34].

To date, *Sinularia levi* (*S*. *levi*) is one of *Sinularia* species that has not been chemically studied. Therefore, we were motivated to carry out this research work in order to report the chemical and biological investigation of this marine invertebrate. The study involved metabolomics profiling, isolation, structure elucidation and biological assessment of the purified metabolites along with protein-protein interaction network construction and analysis followed by molecular docking evaluation.

## Materials and methods

### General experimental procedures

El-Nasr Company for Pharmaceuticals and Chemicals, Egypt is the supplier of the solvents used in this work, e.g., petroleum ether (pet. ether; b.p. 60–80°C), dichloromethane (DCM), ethyl acetate (EtOAc), and methanol (MeOH) which were distilled before use. Deuterated dimethyl sulfoxide (DMSO-*d*_6_) and chloroform (CDCl_3_) (Sigma-Aldrich, Germany), were used for nuclear magnetic resonance (NMR) spectroscopic analyses. Silica gel 60 (El-Nasr Company for Pharmaceuticals and Chemicals, Egypt; 60–120 mesh) was used to perform column chromatography (CC), while silica gel GF_254_ for thin layer chromatography (TLC) (El-Nasr Company for Pharmaceuticals and Chemicals, Egypt) was employed for vacuum liquid chromatography (VLC). Pre-coated silica gel 60 GF_254_ plates (E. Merck, Darmstadt, Germany; 20 × 20 cm, 0.25mm in thickness) was used to carry out TLC analyses and the spots were visualized by spraying with 10% sulfuric acid in methanol followed by heating at 110°C on a hot plate.

The VLC technique was performed on dry silica gel for TLC packed column in the room temperature and then the sample was loaded as solute and the elution was produced by the aid of water vacuum pump.

### Animal material

*S*. *levi* soft coral was collected from a long patchy reef named Ahia Reefs, at the north of Hurghada (Red Sea) at 5 km depth, latitude 27^ο^17’01.0"N and longitude 33^ο^46’21.0" E. Dr. El-Sayed Abd El-Aziz (Department of Invertebrates Lab., National Institute of Oceanography and Fisheries, Red Sea Branch, 84511 Hurghada, Egypt) approved the collection of the soft coral at the field site and has identified the sample as *S*. *levi*.

### Metabolomics analysis

The metabolomics profiling of *S*. *levi* total extract was carried out according to Abdelhafez et al. 2018 [35], using an Acquity Ultra Performance Liquid Chromatography system coupled to a Synapt G2 HDMS quadrupole time-of-flight hybrid mass spectrometer (Waters, Milford, USA). By using Ms converter software, the raw data was converted into divided positive and negative ionization files. Then, the data mining software MZmine 2.10 (Okinawa Institute of Science and Technology Graduate University, Japan) was used for deconvolution, peak picking, alignment, deisotoping, and formula prediction. The detected compounds were finally annotated by comparison with DNP (2020) and METLIN (2020) databases [36–38].

### Extraction and isolation

The collected marine soft coral *S*. *levi* was cut into small pieces and then extracted by maceration with a 50/50 mixture of DCM and methanol. The extracting solution was concentrated under reduced pressure to give solvent-free residue, total extract (120 g). The total extract was suspended in 100 mL of distilled water, to perform liquid-liquid fractionation with pet. ether, followed by EtOAc (300 mL × 6), affording 30.0 and 2.0 g respectively, after evaporating the solvents separately under vacuum. Finally, the remaining mother liquor was concentrated under reduced pressure to afford the aqueous fraction.

The pet. ether fraction of the soft coral *S*. *levi* was subjected to fractionation using VLC (6 × 30 cm, 200 g) technique. It was eluted initially with pet. ether and then the polarity was increased gradually by 10% with EtOAc till the ratio of pet. ether to EtOAc was 60:40. Each polarity was collected and concentrated under reduced pressure affording five subfractions F-I:F-V. The second subfraction F-II (5.0 g) was further fractionated using VLC (3 × 15 cm, 80.0 g) with gradient elution of pet. ether-EtOAc affording four subfractions F-II.a:F-II.d. The subfraction F-II.b (1.0 g) was rechromatographed on a silica gel CC (Ф = 1 mm, L = 70 cm) and eluted with pet. ether-EtOAc gradient mixtures affording five subfractions F-II.b.1- F-II.b.5. Whereas, compound **21** (70.0 mg) was obtained from F-II.b.3 rechromatographing on silica gel CC, using isocratic elution of pet. ether-EtOAc (92:8). Likewise, the subfraction F-II.c was rechromatographed, using pet. ether-EtOAc gradient mixtures on a silica gel column chromatography (CC), yielding compounds **22** (20.0 mg) and **23** (5.0 mg). The subfraction F-II.d was subjected to silica gel CC, which was gradiently eluted with mixtures of pet. ether-EtOAc to afford compounds **24** (23.0 mg) and **25** (19.0 mg).

**Prostantherol** (**21**) Obtained as oily residue. Selected ^1^H-NMR (500 MHz, CDCl_3_) δ_H_: 1.03 (3H, s, H-8), 0.83 (3H, s, H-9), 1.16 (3H, s, H-10), 0.91 (3H, d, *J* = 7.5 Hz, H-11). ^13^C-NMR (125 MHz, CDCl_3_) δ_C_: 18.4 (C-1), 28.7 (C-1a), 18.8 (C-2) 25.7 (C-3), 58.0 (C-3a), 74.4 (C-4), 37.7 (C-5), 29.1 (C-6), 38.4 (C-7), 39.7 (C-7a), 22.3 (C-7b), 28.6 (C-8), 16.1 (C-9), 32.1 (C-10), 16.4 (C-11) [39].

**(24*S*)-24-Methylcholesterol** (**22**) Obtained as white needle crystals. Selected ^1^H-NMR spectral data (600 MHz, CDCl_3_) δ_H_: ^1^H NMR (400 MHz, CDCl_3_) *δ*_H_: 3.50 (1H, m, H-3), 5.32 (1H, brd, *J* = 4.7 Hz, H-6), 0.65 (3H, s, H-18), 0.98 (3H, s, H_3_-19), 0.90 (3H, d, *J* = 6.6 Hz, H-21), 0.76 (3H, d, *J* = 6.8 Hz, H-26), 0.83 (3H, d, *J* = 6.8 Hz, H-27) and 0.75 (3H, d, *J* = 6.8 Hz, H-28). ^13^C-NMR spectral data (CDCl_3_, 100 MHz) δ_C_: 37.4 (C-1), 31.8 (C-2), 71.9 (C-3), 42.5 (C-4), 140.9 (C-5), 121.9 (C-6), 32.1 (C-7), 32.1 (C-8), 50.3 (C-9), 36.7 (C-10), 21.3 (C-11), 39.9(C-12), 42.5 (C-13), 56.9 (C-14), 24.5 (C-15), 28.4 (C-16), 56.2 (C-17), 12.1 (C-18), 19.6 (C-19), 36.4 (C-20), 19.1 (C-21), 33.9 (C-22), 30.8 (C-23), 39.3 (C-24), 31.7 (C-25), 17.8 (C-26), 20.7 (C-27) and 15.6 (C-28) [40].

**Gorgosterol** (**23**) Obtained as white amorphous powder. Selected ^1^H-NMR spectral data (400 MHz, CDCl_3_) *δ*_H_: 3.50 (1H, m, H-3), 5.33 (1H, brd, *J* = 4.7 Hz, H-6), 0.65 (3H, s, H-18), 0.98 (3H, s, H_3_-19), 0.90 (3H, d, *J* = 6.6 Hz, H-21), 0.76 (3H, d, *J* = 6.8 Hz, H-26), 0.83 (3H, d, *J* = 6.8 Hz, H-27) and 0.75 (3H, d, *J* = 6.8 Hz, H-28). ^13^C-NMR spectral data (CDCl_3_, 100 MHz) δ_C_: 37.5 (C-1), 31.8 (C-2), 72.1 (C-3), 42.4 (C-4), 140.9 (C-5), 121.9 (C-6), 32.1 (C-7), 32.2 (C-8), 50.4 (C-9), 36.7 (C-10), 21.3(C-11), 39.9 (C-12), 42.5 (C-13), 56.2 (C-14), 24.5 (C-15), 28.4 (C-16), 58.1 (C-17), 12.1 (C-18), 19.6 (C-19), 35.5 (C-20), 21.3 (C-21), 32.3 (C-22), 25.1 (C-23), 51.0 (C-24), 31.7 (C-25), 21.7 (C-26), 22.3 (C-27), 15.6 (C-28), 14.3 (C-29) and 21.5 (C-30) [40].

**Sarcophine** (**24**) Obtained as colorless crystals, Selected ^1^H-NMR spectral data (500 MHz, DMSO-*d*_6_) δ_H_: 5.76 (1H, dd, *J* = 10, 1.6, H-2), 4.97 (1H, dd, *J* = 10, 1.1, H-3), 1.72, 1.18, and 1.54, (each 3H, s, H-17, 19, and 20, respectively), 1.81 (3H, d, *J* = 1.2, H-18), and 5.07 (1H, brdd, *J* = 8.7, 5.8, H-11). ^13^C-NMR spectral data (DMSO-*d*_6_, 125 MHz) δ_C_: 163.4(C-1), 78.6 (C-2), 120.4(C-3), 143.9 (C-4), 36.5(C-5), 24.8(C-6), 60.5(C-7), 59.3(C-8), 38.7 (C-9), 22.8 (C-10), 124.2(C-11), 135.4(C-12), 36.1 (C-13), 27.0 (C-14), 121.3(C-15), 174.2 (C-16), 8.6(C-17), 16.9(C-18), 15.8(C-19), and 15.1(C-20) [41].

**12-Hydroperoxylsarcoph-10-ene** (**25**) Obtained as amorphous powder, Selected ^1^H-NMR spectral data (500 MHz, DMSO-*d*_6_) δ_H_: 5.62 (1H, dd, *J* = 9.9, 1.6, H-2), 4.91 (1H, br d, *J* = 9.1, H-3), 2.56 (1H, dd, *J* = 6.9, 4.6, H-8), 5.07 (1H, brdd, *J* = 8.7, 5.8, H-11), 1.72, 1.18, and 1.54, (each 3H, s, H-17, 19, and 20, respectively), 1.81 (3H, d, *J* = 1.2, H-18). ^13^C-NMR spectral data (DMSO-*d*_6_, 125 MHz) δ_C_: 163.1(C-1), 78.6 (C-2), 120.7 (C-3), 144.3 (C-4), 35.9 (C-5), 23.8 (C-6), 59.7 (C-7), 57.3 (C-8), 38.9 (C-9), 124.6 (C-10), 136.2 (C-11), 83.0 (C-12), 39.1 (C-13), 21.6 (C-14), 121.8 (C-15), 174.2 (C-16), 8.7 (C-17), 15.5 (C-18), 18.4 (C-19), and 22.6 (C-20) [42].

### *In vitro* cytotoxic activity

The evaluation of the cytotoxic activity of the total extract and isolated compounds of *S*. *levi* was evaluated according to Hassan et al. 2019, using MTT assay in comparison with doxorubicin as a positive control. The human three cancer cell lines, human colon carcinoma (Caco-2), human breast cancer (MCF-7), and hepatocellular carcinoma (HepG-2) was obtained from the American Type Culture Collection (ATCC, Manassas, USA). Briefly, the culture cells were seeded in 96 well microtiter plates at a concentration of 1000–2000 cells/well, 100 μL/well. Then, the cells were incubated for 72 h with the compounds to be tested, using Dulbecco’s Modified Eagle Medium (DMEM) as culture medium, which was discarded at the end of the incubation. The cells were fixed with 150 μL cold trichloroacetic acid with 10% final concentration for 1 h at 4°C. After that, spectrophotometrically at 490 nm, the optical density (OD) of each well was measured with an ELISA microplate reader. The percentage of cell survival was calculated by using the following formula: surviving percent = [O.D. (treated cells)/O.D. (control cells)] x100. The IC_50_ values (the concentrations of compound required to produce 50% inhibition of cell growth) were also calculated [43].

### In silico molecular docking

CDK1 X-ray crystal structure was downloaded from the Protein Data Bank (PDB ID: 3HQ0) [44, 45], corrected and 3D protonated at cutoff 15 Å using amber10:EHT forcefield of Molecular Operating Environment (MOE 2014.0901) software. The binding site was selected at the co-crystallized ligand site with a radius of 4.5 Å. Then molecular docking was performed using Triangle Matcher, London dG, GBVI/WSA as the placement, rescoring function 1 and 2, respectively, as the docking algorithm. The tested meatbolites were drawn using Chemdraw Ultra 12.0, and then transferred as smiles to MOE builder window. Their hydrogens were added and the energy was minimized at the same forcefield.

## Results and discussion

### Metabolomics profiling of the soft coral *S*. *Levi*

**Fig 1:**
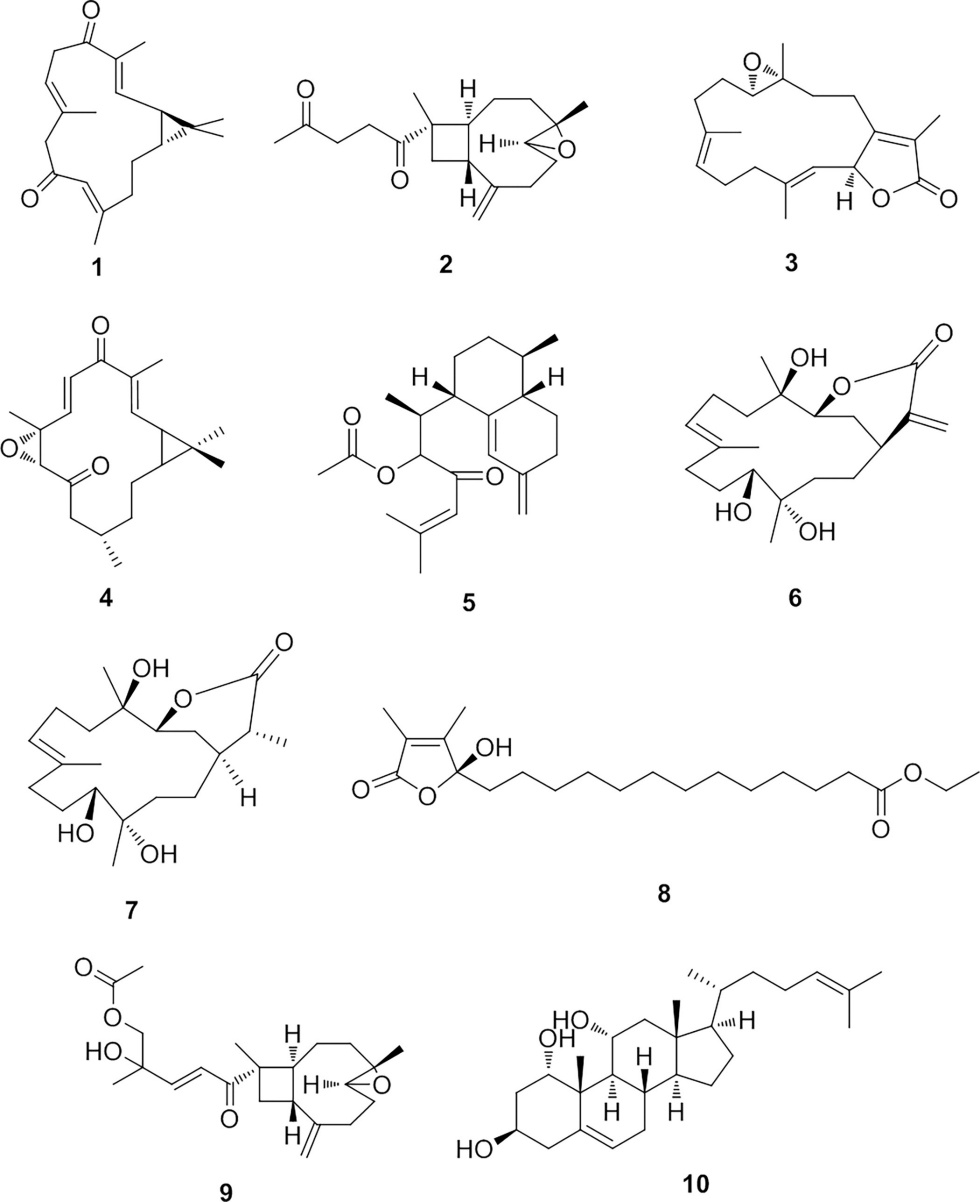
Chemical structures of the dereplicated metabolites (1–10) from *S*. *levi*.

**Fig 2:**
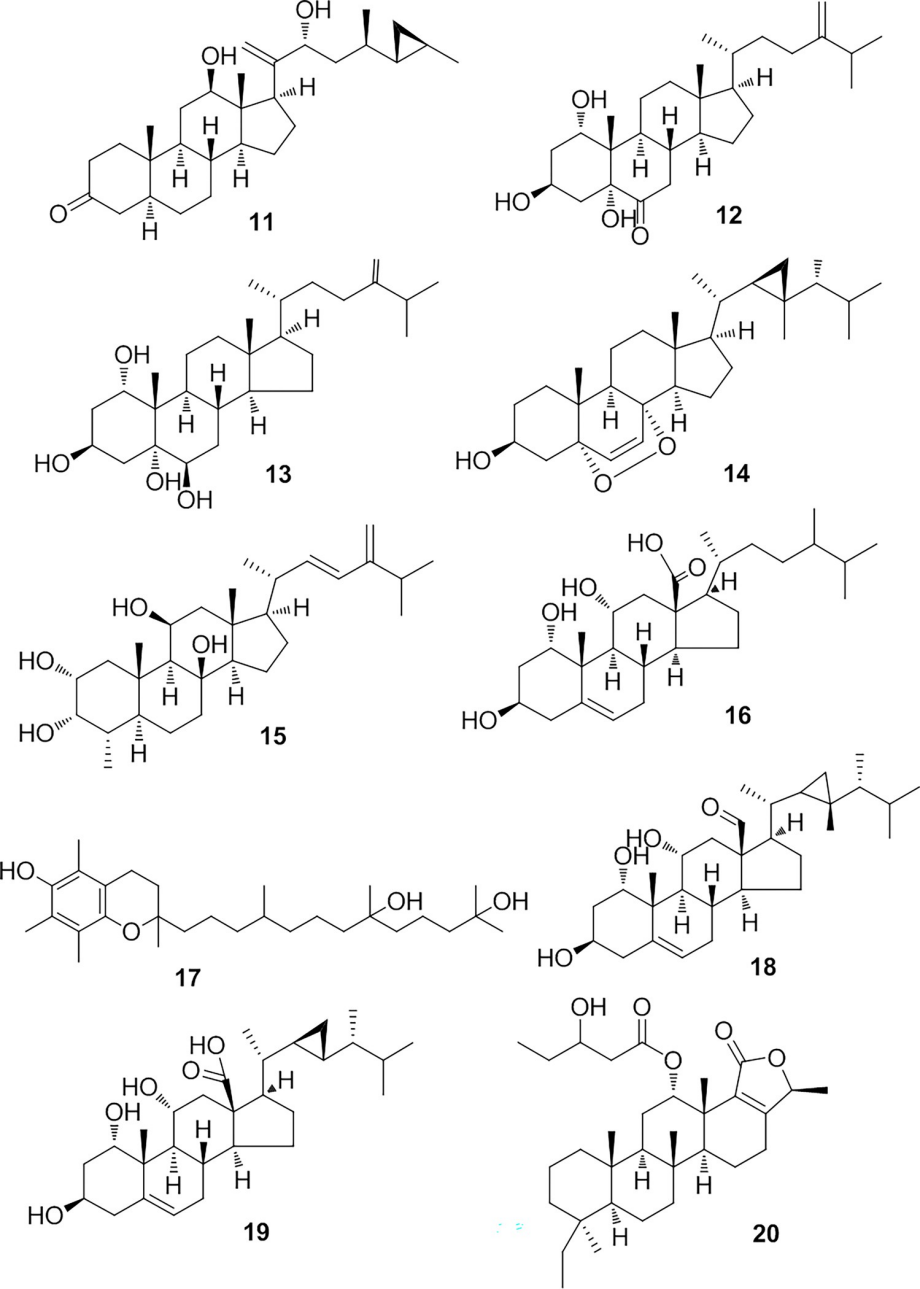
Chemical structures of the dereplicated metabolites (11–20) from *S*. *levi*.

Metabolomics analysis of *S*. *levi* utilizing liquid chromatography-mass spectrometry (LC-MS) based metabolomics approach has resulted in the identification of a number of various secondary metabolites, among which diterpenoids and steroids were predominant (Table 1, Figs 1 and 2). The detected compounds were tentatively identified via searching some databases, e.g., Dictionary of Natural products (DNP) and Marinlit. In this context, the mass ion peak at m/z 301.2168 [M+H]^+^ for the predicted molecular formula C_20_H_28_O_2_ was characterized as 1-epi-10-oxodepressin (**1**). This casbane diterpenoid was previously isolated from the soft coral *S*. *depressa* [19]. Additionally, the mass ion peak at m/z 305.2116 [M+H]^+^ for the suggested formula C_19_H_28_O_3_ was characterized as gibberosin A (**2**), a β-caryophyllene-derived sesquiterpenoid formerly obtained from *S*. *gibberosa* [46]. Likewise, a diterpene with the molecular formula C_20_H_28_O_3_ was dereplicated as isosarcophine (**3**) and/or microclavatin (**4**) from the mass ion peak at m/z 317.2118 [M+H]^+^. The former was previously reported from *S*. *mayi* [47], whereas the latter was formerly purified from *S*. *microclavata* [25]. Moreover, the mass ion peak at m/z 345.2415 [M+H]^+^, in conformity with the predicted molecular formula C_22_H_32_O_3_, was identified as the diterpene sinulodurin B (**5**), earlier purified from *S*. *dura* [48]. Another mass ion peak at m/z 351.2172 [M-H]^-^, corresponding to C_20_H_32_O_5_ was dereplicated as sinuflexolide (**6**). This is another cembranoid compound previously isolated from *S*. *flexibilis* [49]. Similarly, one more cembranoid diterpene was characterized as dihydrosinuflexolide (**7**), in agreement with the mass ion peak at m/z 353.2323 [M-H]^-^ and the molecular formula C_20_H_34_O_5_. This metabolite was also reported from *S*. *flexibilis* [49]. Furthermore, a furanone derivative identified as sinularone I (**8**), in conformity with the mass ion peak at m/z 367.2479 [M-H]^-^ and the molecular formula C_21_H_36_O_5_. This compound was earlier isolated from *Sinularia sp*. [24]. In addition, the mass ion peak at m/z 377.2312 [M+H]^+^, corresponding to the suggested molecular formula C_22_H_32_O_5_, was characterized as gibberosin H (**9**), a xeniaphyllane diterpene that was previously obtained from *S*. *gibberosa* [50].

**Table 1:** List of tentatively identified metabolites and dereplicated from LC-HR-ESI-MS of the soft coral *S*. *levi*.

Comp.	*m/z*	Retention time (min.)	Molecular weight	Name	Molecular formula	Ref.
**1**	301.2168	10.10	300.2095	1-Epi-10-oxodepressin	C_20_H_28_O_2_	[19]
**2**	305.2116	9.30	304.2043	Gibberosin A	C_19_H_28_O_3_	[46]
**3** **4**	317.2118	8.38	316.2043	Isosarcophine/Microclavatin	C_20_H_28_O_3_	[47] [25]
**5**	345.2415	11.80	344.2343	Sinulodurin B	C_22_H_32_O_3_	[48]
**6**	351.2172	9.34	352.2245	Sinuflexolide	C_20_H_32_O_5_	[49]
**7**	353.2323	9.02	354.2396	Dihydrosinuflexolide	C_20_H_34_O_5_	[49]
**8**	367.2479	8.86	368.2552	Sinularone I	C_21_H_36_O_5_	[24]
**9**	377.2312	9.17	376.2239	Gibberosin H	C_22_H_32_O_5_	[50]
**10**	417.3367	10.46	416.3294	Cholesta-5,24-diene-1α,3β,11α-triol	C_27_H_44_O_3_	[21]
**11**	443.3521	13.67	442.3449	Aragusterol E	C_29_H_46_O_3_	[51]
**12**	447.3456	12.77	446.3383	Trihydroxy-24-methylenecholestan-6-one	C_28_H_46_O_4_	[52]
**13**	447.3467	13.20	448.3539	Numersterol A	C_28_H_48_O_4_	[53]
**14**	455.3518	11.05	456.3591	5α,8α-Epidioxygorgosta-6-en-3β-ol	C_30_H_48_O_3_	[54]
**15**	459.3466	13.43	460.3539	Hyrtiosterol	C_29_H_48_O_4_	[55]
**16**	463.3405	12.65	462.3332	24-Methyl-trihydroxy cholestenoic acid	C_28_H_46_O_5_	[56]
**17**	463.3768	13.10	462.3695	Dihydroxy-tocopherol	C_29_H_50_O_4_	[57]
**18**	471.3467	11.17	472.3539	Dihydroxygorgosterol-13-carbaldehyde	C_30_H_48_O_4_	[56]
**19**	473.3264	11.33	474.3337	1α,11α-Dihydroxy-23-demethylgorgosterol-13-carboxylic acid	C_29_H_46_O_5_	[56]
**20**	513.3578	12.41	514.3650	Phyllofolactone J	C_32_H_50_O_5_	[58]
**21**	223.0972	5.01	222.0899	Prostantherol	C_15_H_26_O	[39]
**22**	401.3414	11.86	400.3342	24-Methylcholesterol	C_28_H_48_O	[59]
**23**	425.3573	12.65	426.3645	Gorgosterol	C_30_H_50_O	[59]
**24**	315.2528	8.48	316.2601	Sarcophine	C_20_H_28_O_3_	[60]
**25**	349.1997	7.91	348.1924	12-Hydroperoxylsarcoph-10-ene	C_20_H_28_O_5_	[42]

**Fig 3:**
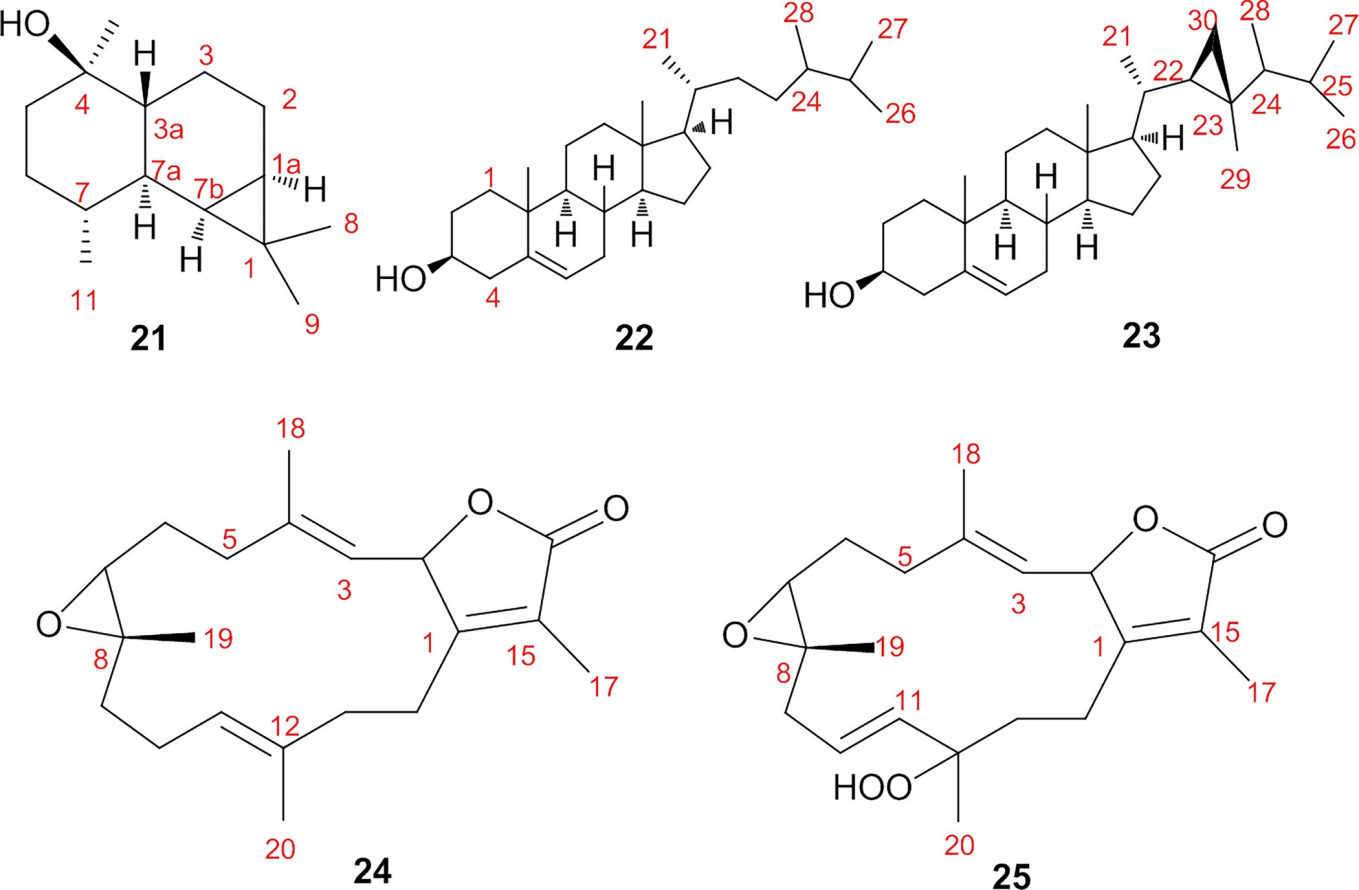
Chemical structures of isolated secondary metabolites (21–25) from *S*. *levi*.

Beside the above-mentioned terpenoids, metabolomics analysis of *S*. *levi* demonstrated that this soft coral was a bountiful source of steroidal constituents. In this regard, a polyoxygenated steroid was dereplicated as cholesta-5,24-diene-1*α*,3*β*,11*α*-triol (**10**) on account of the detected mass ion peak at *m/z* 417.3367 [M+H]^+^, and in line with the molecular formula C_27_H_44_O_3_. This steroid was formerly reported from *S*. *facile* [21]. Another steroid was identified as aragusterol E (**11**) on the basis of the observed mass ion peak at *m/z* 443.3521 [M+H]^+^, and in accordance with the molecular formula C_29_H_46_O_3_. This 26,27-cyclosterol was earlier isolated from the marine sponge *Xesto spongia* [51]. Moreover, the mass ion peak at *m/z* 447.3456 [M+H]^+^, in alignment with the predicted molecular formula C_28_H_46_O_4_ was dereplicated as trihydroxy-24-methylenecholestan-6-one (**12**), a polyhydroxylated steroid formerly obtained from *Sinularia microclavata* [52]. Another related compound was also identified as numersterol A (**13**) based on the mass ion peak at *m/z* 447.3467 [M-H]^-^ and in consonance with the molecular formula C_28_H_48_O_4_. This is also a polyhydroxylated sterol purified isolated before from *S*. *numerosa* [53]. 5*α*,8*α*-Epidioxygorgosta-6-en-3*β*-ol (**14**), a further sterol previously reported from *Sinularia flexibilis* [54], was also characterized from the mass ion peak at *m/z* 455.3518 [M-H]^-^ in compliance with the predicted formula C_30_H_48_O_3_. Compound at *m/z* 459.3466 [M-H]^-^ was dereplicated as the polyhydroxylated sterol, hyrtiosterol (**15**), with the molecular formula C_29_H_48_O_4_, which was as well isolated from *Sinularia* sp. [55]. One more polyhydroxylated sterol was dereplicated as 24-methyl-trihydroxycholestenoic acid (**16**), affiliated to the mass ion peak at *m/z* 463.3405 [M+H]^+^ and the molecular formula C_28_H_46_O_5_. This sterol was obtained before from *S*. *dissecta* [56]. Furthermore, the mass ion peak at *m/z* 463.3768 [M+H]^+^, in alignment with the suggested molecular formula C_29_H_50_O_4_, was dereplicated as dihydroxy-tocopherol (**17**) which was previously purified from *S*. *mayi* [57]. Additionally, the mass ion peak at *m/z* 471.3467 [M-H]^-^for the predicted molecular formula C_30_H_48_O_4_ was identified asdihydroxygorgosterol-13-carbaldehyde (**18**), that was isolated before from *Sinulariadissecta* [56]. The polyhydroxylated sterol with the molecular formula C_29_H_46_O_5_, was characterized as 1*α*,11*α*-dihydroxy-23-demethylgorgosterol-13-carboxylic acid (**19**) from the mass ion peak at *m/z* 473.3264 [M-H]^-^. It was also purified from *S*. *dissecta* [56]. Alongside the previously stated compounds, metabolomics analysis of *S*. *levi* has also led to the characterization of ascalarane-based sesterterpene compound, phyllofolactone J (**20**), in consonance with the mass ion peak at *m/z* 513.3578 [M-H]^-^ and the molecular formula C_32_H_50_O_5_, which was previously isolated from the sponge *Strepsichordaia aliena* [58]. In addition, five metabolites were latterly isolated and identified (Fig 3). A sesquiterpenoidal compound from the mass ion peak at *m/z* 223.0972 [M+H]^+^ in consistence with the molecular formula C_15_H_26_O, was identified as prostantherol (**21**), which was previously isolated from *Sarcophyton glaucom* [39]. Besides, two steroidal compounds, 24-methylcholesterol (**22**) and gorgsterol (**23**) were concluded from the mass ion peaks at *m/z* 401.3414 [M+H]^+^ and 425.3573 [M-H]^-^, in alignment with the molecular formulas C_28_H_48_O and C_30_H_50_O, respectively, both of which were previously reported in *S*. *intacta* [59]. Likewise, two additional diterpenes, sarcophine (**24**) and 12-hydroperoxylsarcoph-10-ene (**25**) were concluded from the mass ion peaks at *m/z* 315.2528 [M-H]^-^ and 349.1997 [M+H]^+^ which were corresponding to the molecular formula C_20_H_28_O_3_ and C_20_H_28_O_5_, respectively. The former was previously reported from *S*. *polydactyla* [60] and the latter was previously isolated from *Sarcophyton glaucoma* [42]. In view of these results, it is worth mentioning that this is the first report for the compounds **1**–**25** from the soft coral *S*. *levi*.

### Structural characterization of the isolated compounds

The mixture of DCM and methanol (v/v = 1/1) total extract of *S*. *levi* was fractionated with different organic solvents with increasing the polarity. The phytochemical study of petroleum (pet.) ether fraction was performed through using chromatographic techniques led to the isolation and identification of five compounds (Fig 3). The isolated compounds were identified using various 1D and 2D NMR spectroscopic methods (S1-S21 Figs in S1 File), including one sesquiterpne, prostantherol (**21**) [39], two steroids; 24-methylcholesterol (**22**) [40] and gorgosterol (**23**) [40], together with two diterpenes; sarcophine (**24**) [41] and 12-hydroperoxylsarcoph-10-ene (**25**) [42]. Interestingly, compounds **21** and **25** were reported for the first time in *Sinularia* genus, on the other hand, it is the first report of compounds **22**, **23**, and **24** in *S*. *levi* species.

### Cytotoxic activity

The cytotoxic potential of the *S*. *levi* total extract (SLTE) was evaluated against three cancer cell lines, human colon carcinoma (Caco-2), human breast cancer (MCF-7), and hepatocellular carcinoma (HepG-2), by using MTT assay in comparison with doxorubicin as a positive control. Whereas, SLTE exhibited potent *in-vitro* multivariate anti-proliferative effects against the tested three cell lines. The most potent activity was against Caco-2 cell line with IC_50_ 3.3 μg/mL, followed by the effect against MCF-7 with IC_50_ 6.4 μg/mL and 8.5 μg/mL toward HepG-2. The *in-vitro* anti-proliferative potency of SLTE was suggested from the US NCI (National Cancer Institute) guidelines, in which all the recorded IC_50_ values did not exceed 20 μg/mL [36] (Table 2).

**Table 2:** Cytotoxic potential of *S*. *levi* total extract and isolated metabolites against cancer cell lines.

Sample	IC_50_ values (mean ± S.E.M; μg/mL)		
	HepG-2	MCF-7	Caco-2
**Total extract**	8.5 ± 0.12	6.4 ± 0.08	3.3 ± 0.06
**21**	22.81 ± 0.42	29.42 ± 0.38	31.14 ± 0.91
**22**	29.22 ± 0.33	32.38 ± 0.52	36.18 ± 0.28
**23**	15.75 ± 0.29	17.35 ± 0.38	18.28 ± 0.26
**24**	26.27 ± 0.18	16.91 ± 0.12	17.44 ± 0.19
**25**	2.13 ± 0.09	3.54 ± 0.07	5.67 ± 0.08
**Doxorubicin**	1.32 ± 0.06	1.72 ± 0.03	2.12 ± 0.04

Literature survey revealed the anti-proliferative *in-vitro* potential of many of the identified and dereplicated secondary metabolites of SLTE. Sinulodurin B (**5**) showed anti-proliferative potency with IC_50_ range of 20−30 μM against malignant +SA mammary epithelial cells, and also exhibited anti-invasive activity in the spheroid disaggregation assay against the human metastatic prostate cancer PC-3M-CT+ cell lines [48]. While, sinuflexolide (**6**) showed cytotoxic activity against the growth of a panel of cell lines, A549, HT-29, KB, and P-388 cells, and dihydrosinuflexolide (**7**) was reported to have selective activity toward the growth of P-388 cells [61]. Likewise, the cytotoxic potential evaluation of the isolated compounds (**21**–**25**) revealed that 12-hydroperoxylsarcoph-10-ene (**25**) exhibited the highest potency against the three tested cell lines (HepG-2, MCF-7 and Caco-2) with IC_50_ 2.13 ± 0.09, 3.54 ± 0.07 and 5.67 ± 0.08 μg/mL, respectively, followed by gorgosterol (**23**) which exhibited IC_50_ 15.75 ± 0.29, 17.35 ± 0.38 and 18.28 ± 0.26 μg/mL, respectively. On the other hand, Sarcophine (**24**) showed potent activity against MCF-7 and Caco-2 with IC_50_ 16.91 ± 0.12 and 17.44 ± 0.19 μg/mL, respectively. The reported data revealed the multivariate effects of 12-hydroperoxylsarcoph-10-ene (**25**), as well it can act as a promising inhibitor of cytochrome P4501A and a good inducer of glutathione-*S*-transferase and quinone reductase [42].

### Construction of protein-protein interaction (PPI) network

**Fig 4:**
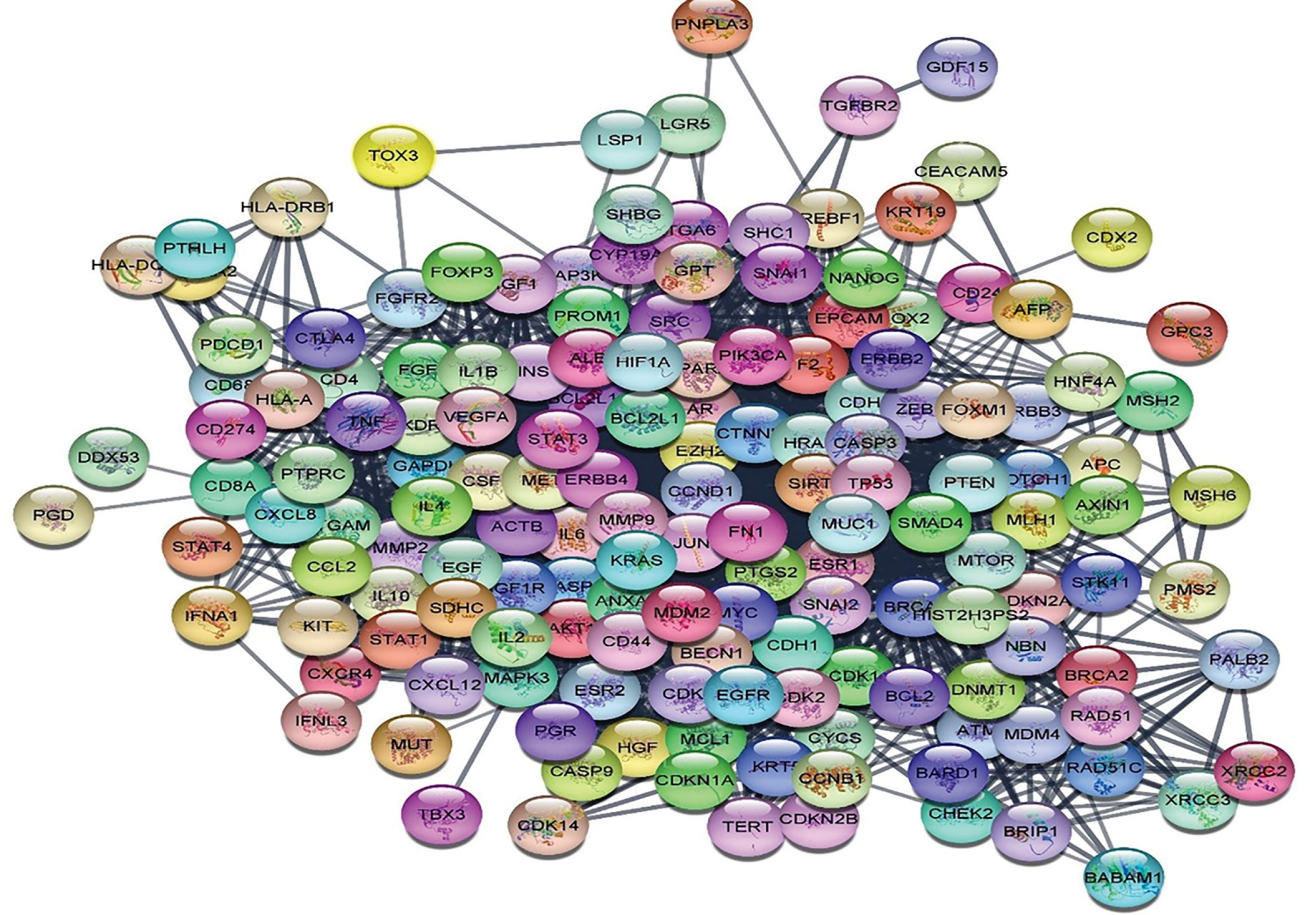
Merged network resulted from the three networks of hcc, colon and breast cancer with organic layout, in which nodes represent protein targets, and the edges represent protein–protein interactions.

Using Cytoscape 3.9.1 software (https://www.cytoscape.org/) [62] and by lunching STRING disease query tool incorporated in it which retrieves network for the top human proteins associated with the queried disease from a weekly updated web source of diseases database (https://string-db.org/) [63] choosing “hepatocellular carcinoma”(hcc), “breast cancer” and “colon cancer”as the word for search and selecting “Homo sapiens” as the type of species. The confidence score was set to score 0.7 and choosing the default setting for the rest of the parameters to achieve three PPI network. Then the three networks merged in one network to achieve the intersected nodes that possessed in the three analyzed networks, and the created network comprised of 157 nodes and 1860 edges (Fig 4) [64].

### Hub gene expression analysis

**Fig 5:**
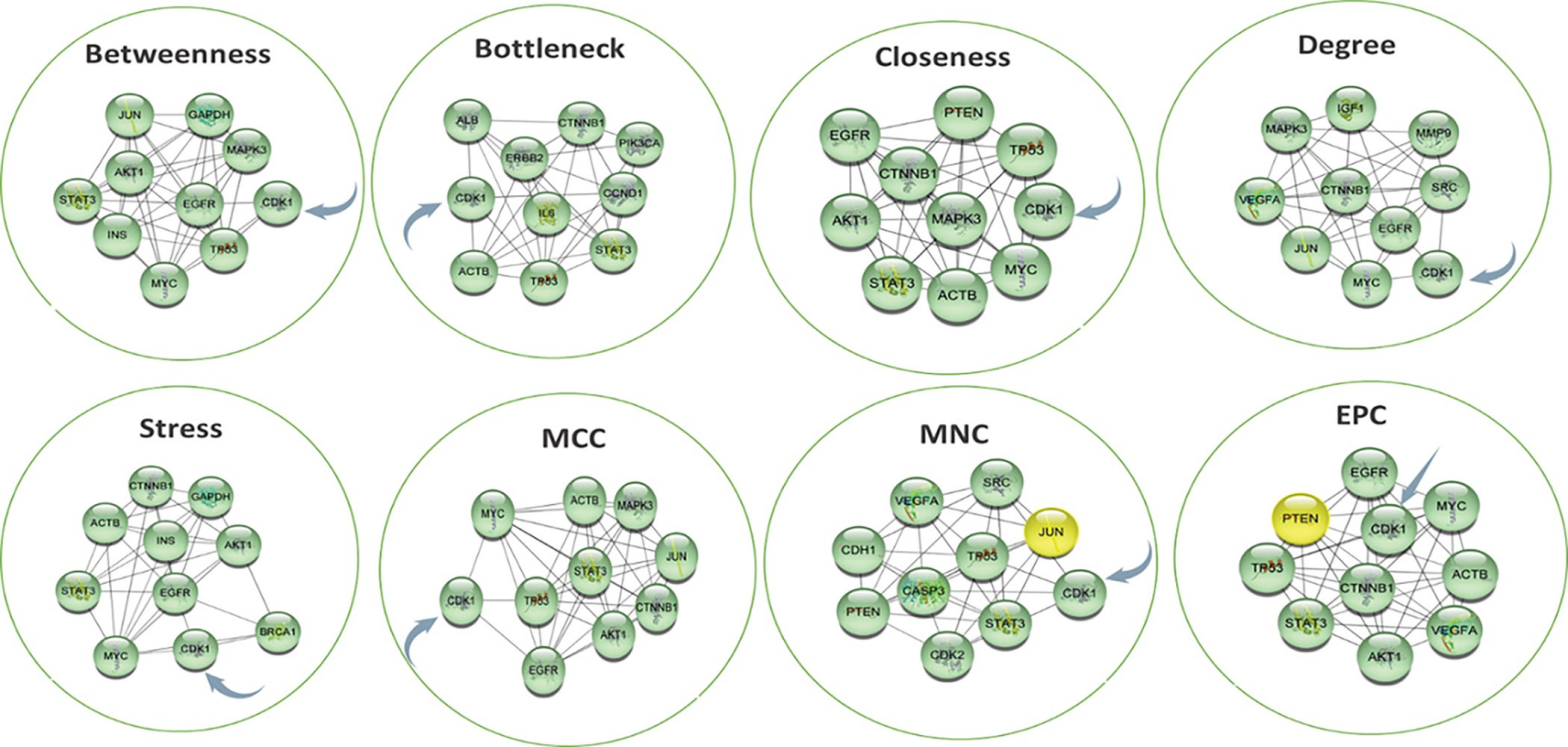
The occurrence of CDK1 in analysis methods of cytoHubba.

The *cytoHubba* plugin Cytoscape is considered a useful exploring interface for the most important nodes in the PPI networks. It used to determine the hub genes in the merged network using ranking methods like (degree, edge percolated component (EPC), maximum neighborhood component (MNC), density of maximum neighborhood component (DMNC), maximal clique centrality (MCC), bottleneck, eccentricity, closeness, radiality, betweenness, stress, and clustering coefficient) [65, 66]. The results shown in Table 3 demonstrated that 14 nodes repeated in more than two analysis methods, regarding the occurrence, and CDK1 possessed the highest score as it appeared in 8 methods from the 12 methods, followed by STAT3, EGFR, CTNNB1, and MYC with score of 6 for each (Fig 5).

**Table 3:** List of the protein coding genes present in at least two methods from twelve different methods of the *cytoHubba* plugin Cytoscape.

No.	Node	Occurence
1	CDK1	8
2	STAT3	6
3	EGFR	6
4	CTNNB1	6
5	MYC	6
6	Tp53	5
7	Akt1	5
8	ACTB	5
9	MAPK3	4
10	JUN	3
11	INS	2
12	GAPDH	2
13	PTEN	2
14	VEGFA	2

### *In silico* molecular docking

One of the possible molecular targets of *Sinularia* metabolites to inhibit cancerous cell growth is cyclin-dependent kinase 1 (CDK1) inhibition. *Sinularia* metabolites including; sinularin, 5-epi-sinuleptolide and numersterol A have been previously reported to inhibit tumor growth through cell cycle arrest and inhibiting its regulating enzyme CDK1 [67–69].

**Fig 6:**
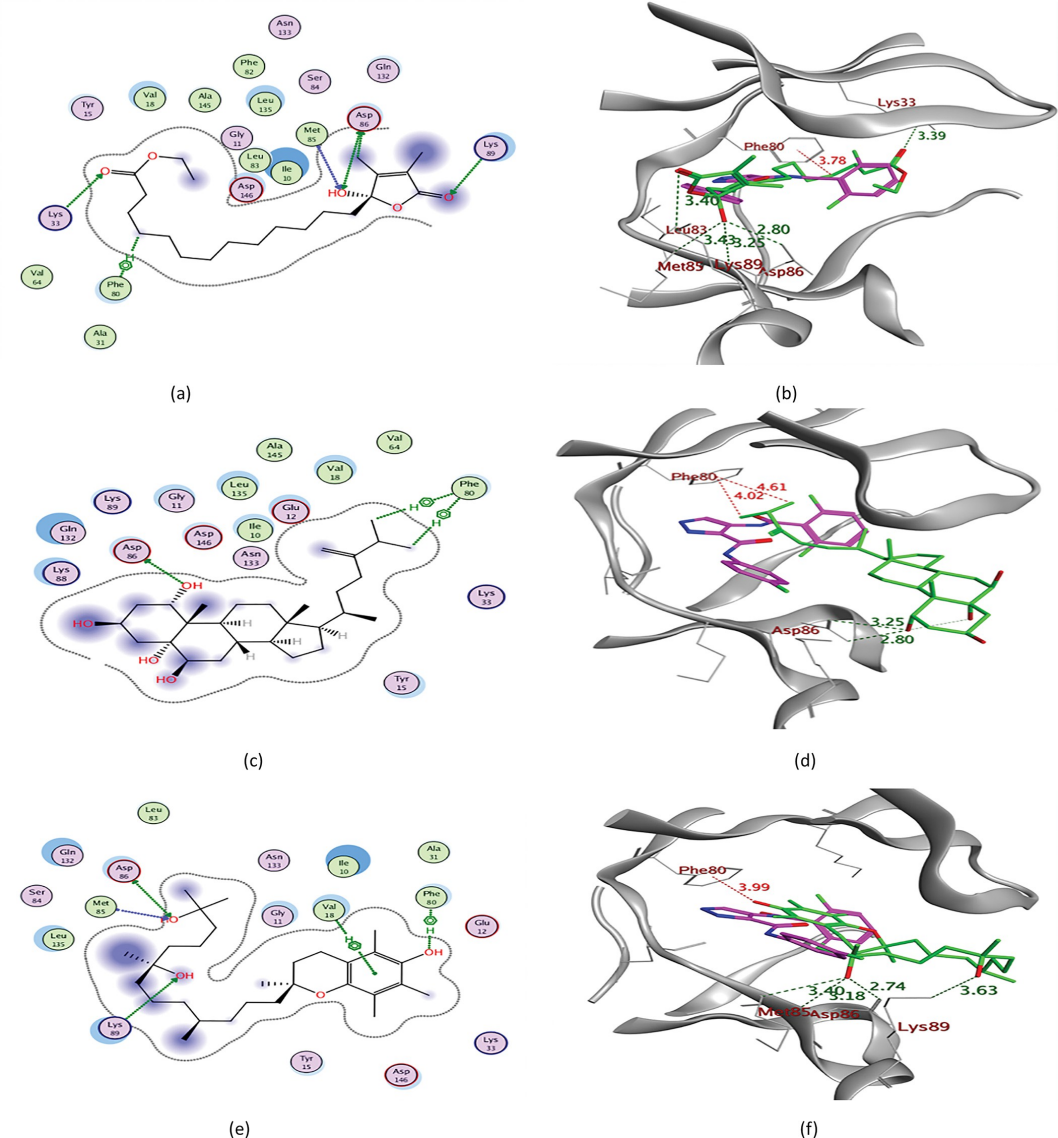
The 2D and 3D interaction patterns of the best orientation of metabolites 8 (a, b), 13 (c, d) and 17 (e, f) to CDK1 (PDB: 5HQ0) showing as green stick model relative to the magenta co-crystallized ligand. The formed hydrogen bonds and hydrophobic interactions appeared as green and red dotted lines, respectively, with their corresponding lengths in Å.

**Fig 7:**
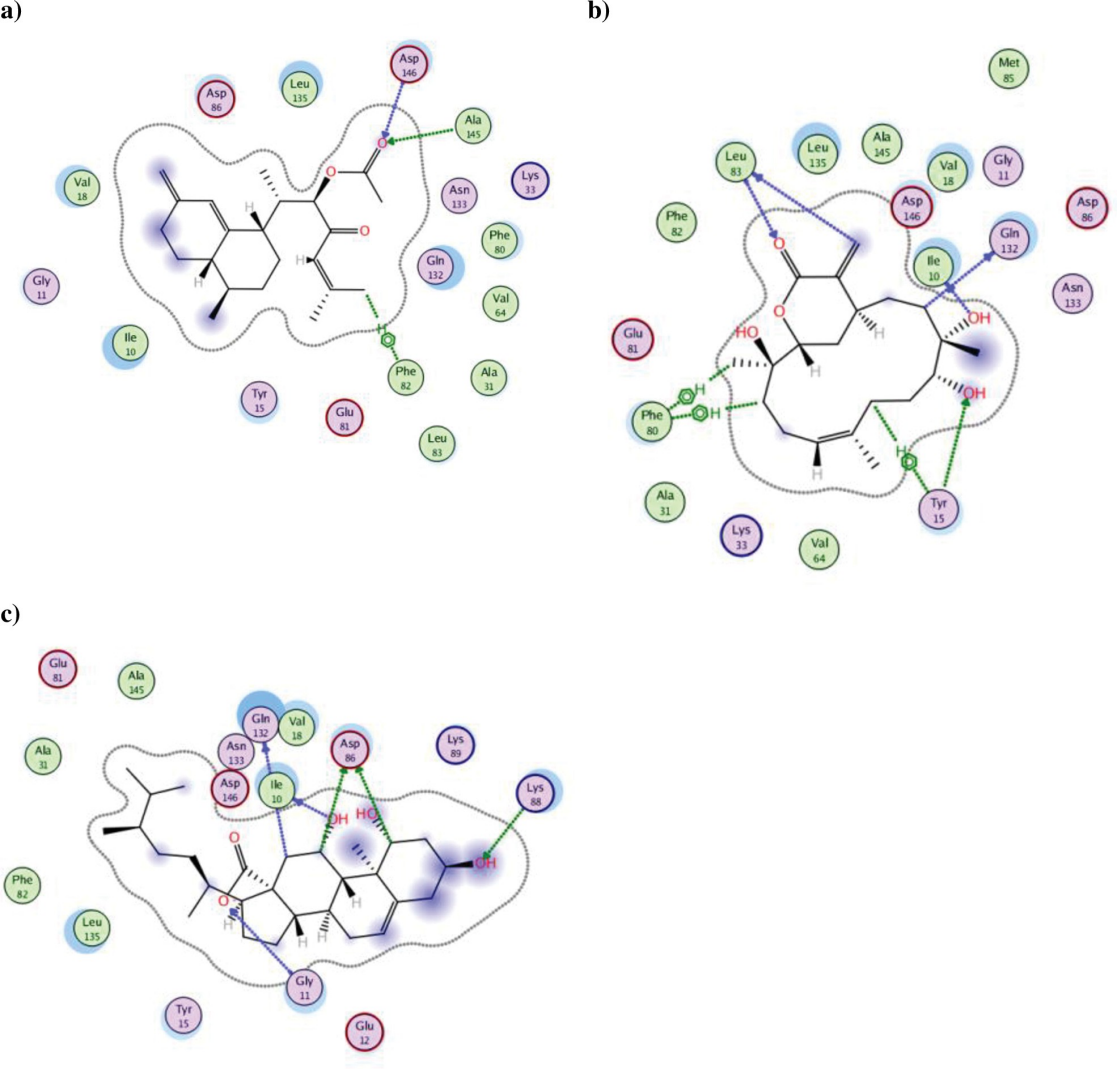
The 2D interaction patterns of the best orientation of metabolites 5 (a), 6 (b) and 16 (c) to the active site residues of CDK1 (PDB: 5HQ0).

CDK1 is a member of the kinase family that binds to cyclin B to initiate the mitosis at the cell cycle M-phase [70]. Additionally, CDK1 binds to cyclin A to ensure complete mitosis of the cell [71, 72]. Establishing the importance of CDK1 inhibition to arrest the cancerous cell cycle, the isolated metabolites **1–25** inhibitory potentials were evaluated using *in silico* molecular docking. The protein crystal structure of CDK1 was retrieved from the Protein Data Bank https://www.rcsb.org/ using PDB:5HQ0 [44, 45], then prepared as mentioned in the methodology section before validating the docking protocol. Molecular docking protocol validation was done by re-docking the co-crystallized ligand **LZ9** to get the lowest possible RMSD of 0.47 and binding energy score of -9.97 kcal/mol. The achieved molecular docking results of the isolated metabolites **1–25** were presented in Table 4 and Figs 6 and 7.

**Table 4:** The molecular docking results of the isolated derivatives 1–25 using CDK1 PDB:5HQ0.

Comp.	Binding energy score (kcal/mol)	Ligand		CDK1			Interaction type	Distance (Å)	H-bond Angle	Energy (kcal/mol)
		Interacting moieties		Interacting moieties	Amino acid residue					
**LZ9**	-9.97	N6	8	O	Leu	83	H-donor	2.92	145.284	-3.3
		N11	14	O	Glu	81	H-donor	2.66	146.518	-6.1
		C26	36	OD1	Asp	86	H-donor	3.30	111.899	-0.7
		N10	13	N	Leu	83	H-acceptor	3.26	124.819	-2.6
**1**	-7.92	C	8	6-ring	Phe	80	H-pi	3.50		-0.3
		C	9	6-ring	Phe	80	H-pi	4.15		-0.6
**2**	-7.53	C	13	OD2	Asp	146	H-donor	3.42	132.649	0.4
		C	19	OD1	Asp	86	H-donor	3.67	132.649	-0.3
		O	22	CB	Asp	86	H-acceptor	3.14	119.81	-0.3
		C	24	6-ring	Phe	80	H-pi	3.82		-0.3
**3**	-9.09	O	23	N	Leu	83	H-acceptor	2.87	133.881	-2.9
		O	23	CB	Leu	83	H-acceptor	3.31	162.663	-0.2
		C	12	6-ring	Phe	80	H-pi	4.00		-0.3
**4**	-8.53	C	19	6-ring	Phe	80	H-pi	3.57		-0.4
**5**	-9.83	O	22	CB	Ala	145	H-acceptor	3.53	148.981	-0.2
		O	22	N	Asp	146	H-acceptor	3.00	148.981	-1.4
		C	18	6-ring	Phe	82	H-pi	4.72		-0.2
**6**	-9.23	C	22	O	Leu	83	H-donor	3.37	134.691	-0.6
		O	24	O	Ile	10	H-donor	3.13	134.691	-1
		C	25	O	Gln	132	H-donor	3.47	156.806	0.1
		O	15	OH	Tyr	15	H-acceptor	3.19	156.806	-0.2
		O	21	N	Leu	83	H-acceptor	3.03	138.188	-3.6
		C	2	6-ring	Tyr	15	H-pi	4.60		-0.2
		C	5	6-ring	Phe	80	H-pi	4.14		-0.2
		C	23	6-ring	Phe	80	H-pi	4.57		-0.2
**7**	-8.54	C	9	O	Ile	10	H-donor	3.11	141.395	-0.4
		O	15	OD2	Asp	146	H-donor	3.10	131.195	-0.6
		O	17	O	Leu	83	H-donor	3.00	150.88	-1.1
		C	22	OD1	Asp	86	H-donor	3.11	140.80	-0.3
		O	17	N	Leu	83	H-acceptor	3.11	115.65	-1.3
**8**	-10.92	O	13	OD1	Asp	86	H-donor	2.8	116.68	-3.4
		O	8	CE	Lys	89	H-acceptor	3.4	121.35	-0.4
		O	13	CA	Met	85	H-acceptor	3.43	141.25	-0.2
		O	13	N	Asp	86	H-acceptor	3.25	140.88	-0.1
		O	26	CE	Lys	33	H-acceptor	3.39	120.18	-0.9
		C	19	6-ring	Phe	80	H-pi	3.78		-0.3
**9**	-7.29	C	10	OD1	Asp	86	H-donor	3.11	103.46	6.1
		O	21	OD2	Asp	146	H-donor	2.94	102.26	-1.7
		C	23	OD1	Asn	133	H-donor	3.14	103.25	-0.3
		C	22	6-ring	Tyr	15	H-pi	4.13		-0.3
		C	29	6-ring	Phe	80	H-pi	4.21		-0.2
**10**	-9.01	O	33	O	Gln	132	H-donor	2.87	106.35	-2
		O	32	CB	Gln	132	H-acceptor	3.09	113.85	-0.3
		C	19	6-ring	Phe	80	H-pi	3.65		-0.4
**11**	-8.95	O	26	OD1	Asp	86	H-donor	3.11	83.82	-0.3
		O	26	CB	Gln	132	H-acceptor	3.25	93.92	-0.2
		O	31	OH	Tyr	15	H-acceptor	2.82	147.57	-0.2
		C	33	6-ring	Phe	80	H-pi	4.88		-0.4
**12**	-8.74	O	31	OD2	Asp	86	H-donor	3.41	140.81	-0.5
		O	32	NZ	Lys	88	H-acceptor	3.28	119.39	-0.4
		C	26	6-ring	Phe	80	H-pi	3.94		-0.3
**13**	-10.38	O	19	OD1	Asp	86	H-donor	3.25	151.98	-0.5
		O	19	OD2	Asp	86	H-donor	2.8	154.93	-2.5
		C	29	6-ring	Phe	80	H-pi	4.02		-0.4
		C	30	6-ring	Phe	80	H-pi	4.61		-0.2
**14**	-7.31	O	20	NZ	Lys	88	H-acceptor	3.17	147.03	-1.5
		O	22	CB	Gln	132	H-acceptor	3.01	132.32	-0.4
**15**	-7.91	C	13	O	Gln	132	H-donor	3.41	155.72	0.7
		C	14	OD1	Asp	86	H-donor	3.38	102.49	-0.3
		O	27	N	Glu	12	H-acceptor	2.86	141.78	-1.3
		O	28	NZ	Lys	88	H-acceptor	3.08	143.42	-3.5
**16**	-9.16	C	6	OD1	Asp	86	H-donor	3.03	155.72	-0.3
		C	13	O	Gln	132	H-donor	3.43	102.49	1.1
		C	14	OD1	Asp	86	H-donor	3.31	141.78	-0.3
		O	22	O	Ile	10	H-donor	2.7	143.42	-1.3
		O	23	NZ	Lys	88	H-acceptor	3.3	123.95	-2.4
		O	33	CA	Gly	11	H-acceptor	3.09	125.86	-0.3
**17**	-11.39	O	33	OD1	Asp	86	H-donor	2.74	160.14	-2.9
		O	31	NZ	Lys	89	H-acceptor	3.63	173.031	-0.3
		O	33	CA	Met	85	H-acceptor	3.4	149.352	-0.3
		O	33	N	Asp	86	H-acceptor	3.18	163.14	0.6
		O	25	6-ring	Phe	80	H-pi	3.99		-0.6
		6-ring	CG1	Val	18	pi-H	4.12	-0.5		
		6-ring	CG2	Val	18	pi-H	3.82	-0.2		
**18**	-8.86	C	13	O	Gln	132	H-donor	3.48	145.19	-0.3
		C	14	O	Ile	10	H-donor	3.31	157.84	-0.4
		O	21	OD1	Asp	86	H-donor	3.31	127.48	-0.3
		O	21	OD2	Asp	86	H-donor	2.87	113.22	-1.4
		O	22	OD1	Asp	86	H-donor	2.65	154.76	-1.4
		C	28	6-ring	Phe	80	H-pi	3.66		-0.2
**19**	-6.98	O	22	OD1	Asn	133	H-donor	2.79	128.31	-0.6
		O	21	CB	Asp	146	H-acceptor	3.36	132.57	-0.2
		O	33	N	Glu	12	H-acceptor	2.94	107.92	-1.6
		O	33	OH	Tyr	15	H-acceptor	2.83	109.92	-1.0
		O	34	CA	Gly	11	H-acceptor	2.98	120.36	-0.4
**20**	-6.70	O	33	N	Glu	12	H-acceptor	3.12	142.57	-1.2
		O	25	6-ring	Phe	80	H-pi	3.91		-0.4
**21**	-6.47	C	1	6-ring	Phe	80	H-pi	4.29		-0.5
		O	28	6-ring	Phe	80	H-pi	4.16		-0.8
**22**	-8.54	C	1	6-ring	Phe	80	H-pi	3.36		-0.3
**23**	-8.09	O	45	N	Asp	146	H-acceptor	3.12	142.86	-1
		C	9	6-ring	Phe	80	H-pi	3.43		-0.4
**24**	-7.64	C	17	6-ring	Phe	80	H-pi	3.92		-0.7
**25**	-8.63	O	51	O	Ile	10	H-donor	3.1	128.73	-1.2
		O	41	NZ	Lys	33	H-acceptor	2.85	118.76	-8.1

The best interaction pattern and binding energy that surpassed the co-crystallized ligand was demonstrated by the isolated metabolites sinularone I (**8**), numersterol A (**13**) and dihydroxy-tocopherol (**17**). As illustrated, the furanone derivative sinularone I (**8**) formed H -bond with the conserved catalytic Lys33 by its terminal carbonyl ester with bond length 3.39 Å and 120.18 angle (Fig 6A and 6B). Moreover, its hydroxyl moiety formed two H-bonds with Asp86 near CDK1 pocket entrance and a hydrophobic interaction with the gatekeeper residue Phe80 (Fig 6B) [73] in overall binding energy of -10.92 kcal/mol. On the other hand, one of the hydroxyl group of the sterol backbone of numersterol A (**13**) formed two H-bonds with Asp86 with distance 2.80 Å (angle 154.93) and 3.25 Å (angle 151.98) beside two other hydrophobic interaction with Phe80 with overall binding energy of -10.38 kcal/mol (Fig 6C and 6D). The best binding energy was achieved by the dihydroxy-tocopherol (**17**) with overall binding energy of -11.39 kcal/mol compared with -9.97 kcal/mol of **LZ9**. Moreover, **17** established two short-distanced H-bonds formed with Asp86 of length 2.74 Å (angle 160.14) and 3.18 Å (angle 163.14). Furthermore, **17** formed hydrophobic interaction with the gatekeeper Phe80 and the hydrophobic residue Val18 in addition to other H-bonds with Lys89 and Met85 (Fig 6E and 6F). Comparable binding energy score to **LZ9** was observed with sinulodurin B (**5**), sinuflexolide (**6**) and 24-methyl-trihydroxycholestenoic acid (**16**) giving -9.83, -9.23 and -9.16 kcal/mol, respectively, with many hydrophobic and H-bond interactions with CDK1 pocket residues. The carbonyl of the ester group of sinulodurin B (**5**) established two H-bonds with Ala145 and Asp146 in addition to a hydrophobic interaction with Phe82 by its aliphatic side chain (Fig 7A). At the same time, sinuflexolide (**6**) showed many H-bonds with Leu83, Ile10 and Gln132 beside the hydrophobic interaction with Tyr15 and Phe80 (Fig 7B). Likewise, the 24-methyl-trihydroxycholestenoic acid (**16**) formed several H-bonds with the pocket resides Ile10, Gly11, Asp86, Gln132 and Lys88 with average bond length of 3.0 Å (angle 155.72) (Fig 7C).

It is worth mentioning that most of the isolated metabolites formed hydrophobic interaction with the gatekeeper residue Phe80 except metabolites **5, 7, 14, 15, 16, 19** and **25**. However, 12-hydroperoxylsarcoph-10-ene (**25**) showed tight binding with the catalytic Lys33 with H-bond length of 2.85 Å. Additionally, each of the metabolites isosarcophine (**3**), sinuflexolide (**6**) and dihydrosinuflexolide (**7**) managed to form two H-bonds with the hinge region residue Leu83 with average distance of 3.0 Å (Table 4). Similarly, gibberosin A (**2**), sinulodurin B (**5**), dihydrosinuflexolide (**7**), gibberosin H (**9**) and the polyhydroxylated sterol (**19**) formed H-bonds with the DFG residue Asp146 with average bond length of 3.1 Å. In conclusion, most of the isolated *Sinularia* metabolites showed better interaction pattern with CDK1 crucial residues and/or better binding energy than **LZ9,** which supported the theory of their ability to inhibit the cancerous cell multiplication through CDK1 inhibition.

**Fig 8:**
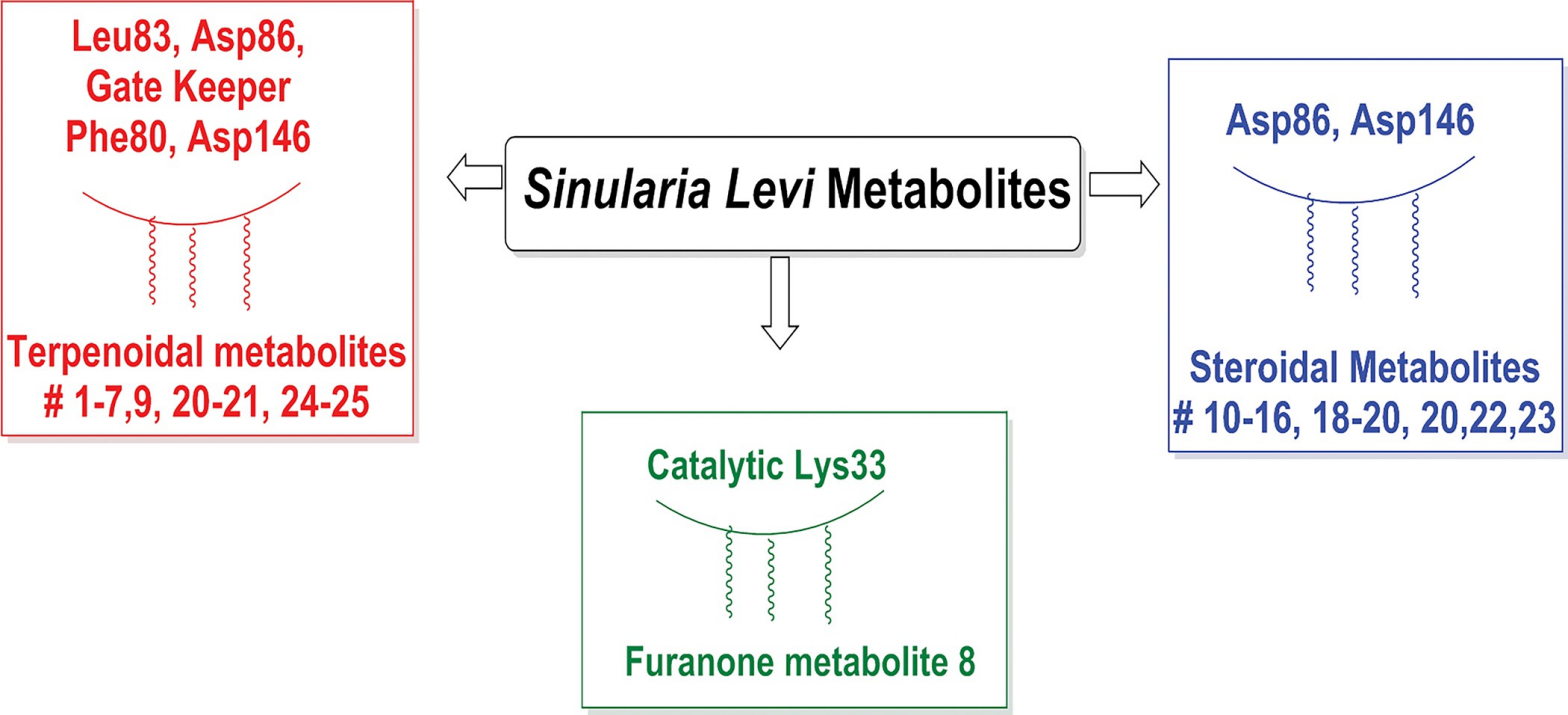
Summary of expected structure actovoty relationship of sinularia metabolites and CDK1 residues.

In summary, most of the sesquiterpene and diterpene metabolites of *S*. *levi* showed H-bonds with the crucial Leu83 and Asp86 in a similar way to the co-crystallized ligand. Moreover, they managed to interact with Asp124 and the gatekeeper Phe80. On the other hand, the furanone metabolite **8** was the only metabolite to hinder Lys33 activity through H-bonding. In the same context, most of the steroidal metabolites were monitored to form H- bonds with the crucial Asp86 and Asp146 (Fig 8).

#### Drug likeness and lead optimization

Based on the outcomes derived from molecular docking investigations, primarily focused on binding interactions rather than energy scoring, compound **17** emerges as a promising candidate for lead optimization processes. Subsequently, an ADME analysis was conducted for compound **17** utilizing the SwissADME tool (S22 Fig in S1 File) [74], yielding insightful information. The compound conforms to Lipinski’s Rule of Five, even though with a singular violation attributed to an MLOGP value exceeding 4.15. Furthermore, it exhibits a favorable bioavailability score of 0.55. Importantly, the structural assessment of compound **17** revealed the absence of PAINS substructures, thereby enhancing its overall desirability. It also displayed a notable absence of Brenk alerts associated with toxicity. Conversely, compound **17** falls short of complying with the Veber rule due to an excess of rotatable bonds exceeding the stipulated threshold. Further, it exhibits suboptimal solubility characteristics.

Based on these findings, coupled with the observed interaction patterns with the CDK1 receptor, several potential modifications can be proposed to enhance the Leadlikeness of compound **17**. One such modification entails the removal of three methyl groups attached to the benzene ring, a modification expected to reduce LogP without disrupting the established interactions. Additionally, optimizing compound **17** involves shortening the aliphatic chain connected to the chromane moiety through the removal of 2 or 3 carbon atoms, concomitantly introducing an olefinic group to simultaneously adjust LogP and decrease the number of rotatable bonds. Another possibility for enhancement of solubility and bioavailability, achievable through O-glycosylation of one or two of the alcoholic hydroxyl groups or the phenolic moiety.

## Conclusions

This study focused on the phytochemical investigation of the soft coral, *S*. *levi*, for the first time. LC-MS based metabolomics has resulted in the dereplication of 25 various secondary metabolites (**1–25**), among which diterpenoids and steroids were predominant. Besides, the use of different chromatographic and spectroscopic techniques (1D and 2D-NMR analysis) resulted in the isolation and identification of five compounds **21**–**25**, including diterpenes, steroids and a sesquiterpene. Notably, this is the first report of compounds (**21**) and (**25**) in *Sinularia* genus. In addition, the results of MTT assay revealed that *S*. *levi* total extract exhibited potent *in-vitro* multivariate anti-proliferative effect against the three tested cell lines; Caco-2, MCF-7 and HepG-2, in comparison with doxorubicin as a positive control. Moreover, protein-protein interaction network was constructed from three networks and subjected to comprehensive bioinformatic evaluation. The top hub protein coding gene was identified as CDK1, which has been identified as a key therapeutic target for the anticancer activity. The following *in silico* molecular docking on CDK1 suggested the possible mechanism responsible for the cytotoxic potential of *S*. *levi* total extract.

## Supporting information

S1 File S1-22 Figs: Spectra of isolated compounds. (DOCX)
