# Supplementary material for: Cytotoxic metabolites from Sinularia levi supported by network pharmacology
Source: PLoS One. 2024 Feb 6;19(2):e0294311. doi: 10.1371/journal.pone.0294311 (PMC10846738; doi:10.1371/journal.pone.0294311)
Supplement: S1 File — (DOCX) [file pone.0294311.s001.docx]

**Cytotoxic Metabolites from *Sinularia levi* Supported by Network Pharmacology**

Mingna Sun,^a†^ Miada F. Abdelwahab,^b†^ Jianye Zhang,^a†^ Mamdouh Nabil Samy,^b^ Nada M. Mohamed,^c^ Islam M. Abdel-Rahman,^d^ Faisal Alsenani,^e^ Usama Ramadan Abdelmohsen ^b,f,^* and Basma Khalaf Mahmoud ^b^

^a^ Guangzhou Municipal and Guangdong Provincial Key Laboratory of Molecular Target & Clinical Pharmacology, the NMPA and State Key Laboratory of Respiratory Disease, School of Pharmaceutical Sciences and the Fifth Affiliated Hospital, Guangzhou Medical University, Guangzhou 511436, China

^b^ Department of Pharmacognosy, Faculty of Pharmacy, Minia University, 61519 Minia, Egypt

^c^ Department of Pharmaceutical Chemistry, Modern University for Technology and Information (MTI), Cairo, Egypt

^d^ Department of Pharmaceutical Chemistry, Faculty of Pharmacy, Deraya University, New-Minia ,61768-Minia, Egypt.

^e^ Department of Pharmacognosy, College of Pharmacy, Umm Al-Qura University, Makkah 21955, Saudi Arabia. [fssenani@uqu.edu.sa](mailto:fssenani@uqu.edu.sa)

^f^ Department of Pharmacognosy, Faculty of Pharmacy, Deraya University, 61111 New Minia, Egypt

^†^ Those authors have equally contributed to this work.

***** Correspondence: E-mail: [usama.ramadan@mu.edu.eg](mailto:usama.ramadan@mu.edu.eg); Fax: +20-086-2369075; Tel: +20-086-2347759

# Abstract

# The *in-vitro* anti-proliferative evaluation of *Sinularia levi* total extract against three cell lines revealed its potent effect against Caco-2 cell line with IC_50_ 3.3 µg/mL, followed by MCF-7 and HepG-2 with IC_50_ 6.4 µg/mL and 8.5 µg/mL, respectively, in comparison to doxorubicin. Metabolic profiling of *S. levi* total extract using liquid chromatography coupled with high-resolution electrospray ionization mass spectrometry (LC-HR-ESI-MS) revealed the presence of phytoconstituents clusters consisting mainly of steroids and terpenoids (1-20), together with five metabolites 21-25, which were additionally isolated and identified through the phytochemical investigation of *S. levi* total extract through various chromatographic and spectroscopic techniques. The isolated metabolites included one sesquiterpene, two steroids and two diterpenes, among which compounds prostantherol (21) and 12-hydroperoxylsarcoph-10-ene (25) were reported for the first time in *Sinularia* genus. The cytotoxic potential evaluation of the isolated compounds revealed variable cytotoxic effects against the three tested cell lines. Compound 25 was the most potent with IC_50_ value of 2.13 ± 0.09, 3.54 ± 0.07 and 5.67 ± 0.08 µg/mL against HepG-2, MCF-7 and Caco-2, respectively, followed by gorgosterol (23) and sarcophine (24). Additionally, network analysis showed that cyclin-dependent kinase 1 (CDK1) was encountered in the mechanism of action of the three cancer types. Molecular docking analysis revealed that CDK1 inhibition could possibly be the reason for the cytotoxic potential.

# Keywords: *Sinularia levi*; metabolomics profiling; terpenoids; sarcophine; cyclin-dependent kinase 1

**Fig S1: Positive ion mode ESI total ion chromatogram of *S. levi* total extract.**

**Fig S2: Negative ion mode ESI total ion chromatogram of *S. levi* total extract.**

**
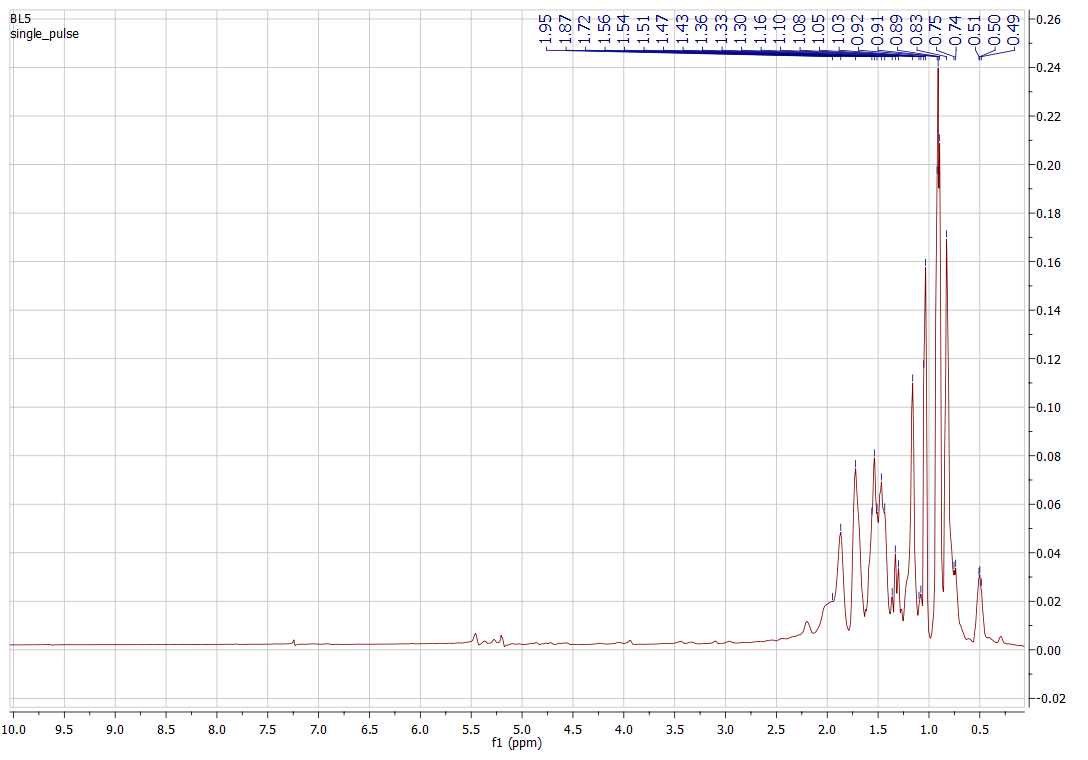
**

**Fig S3**: **^1^H- NMR spectrum of compound 21 (500MHz, CDCl_3_).**

**
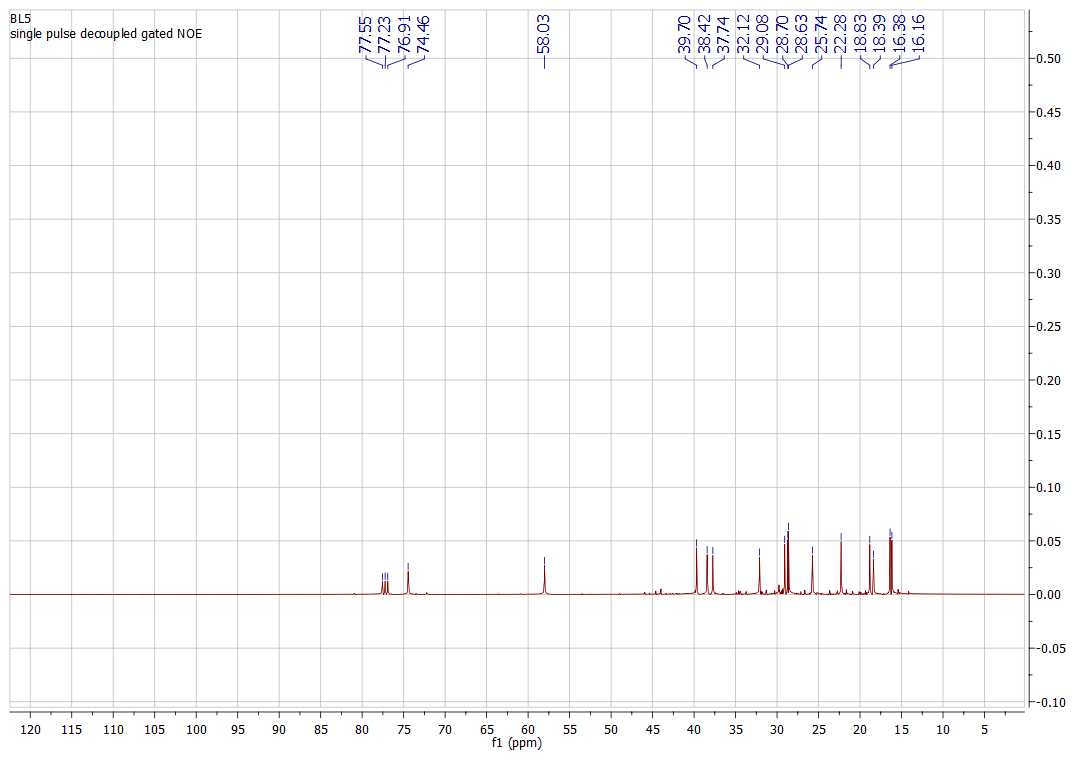
**

**Fig S4**: **^13^C- NMR spectrum of compound 21 (125MHz, CDCl_3_).**


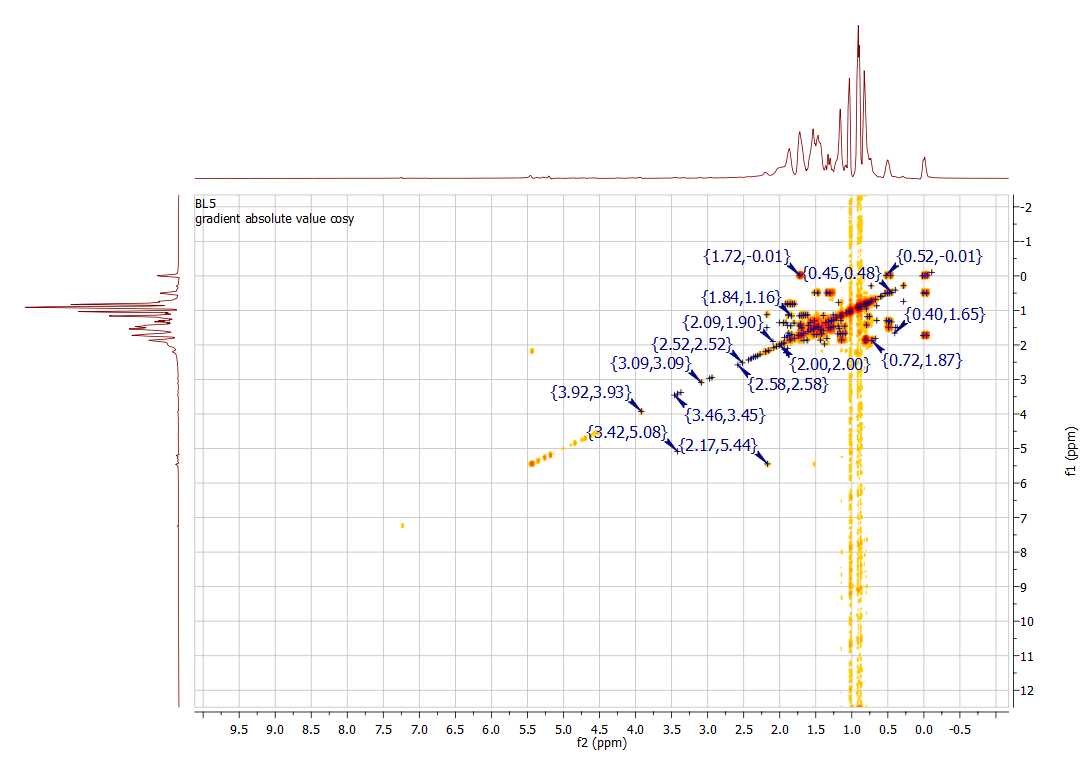


**Fig S5**: **^1^H-^1^H COSY spectrum of compound 21.**


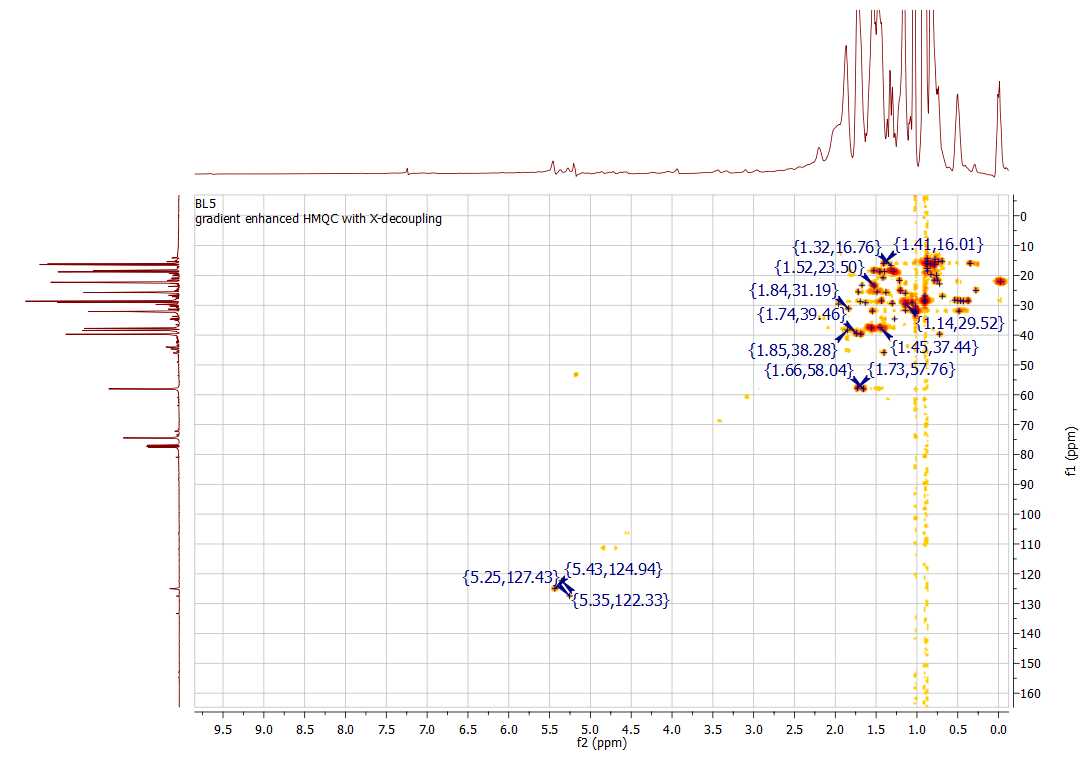


**Fig S6**: **HMQC spectrum of compound 21.**

**
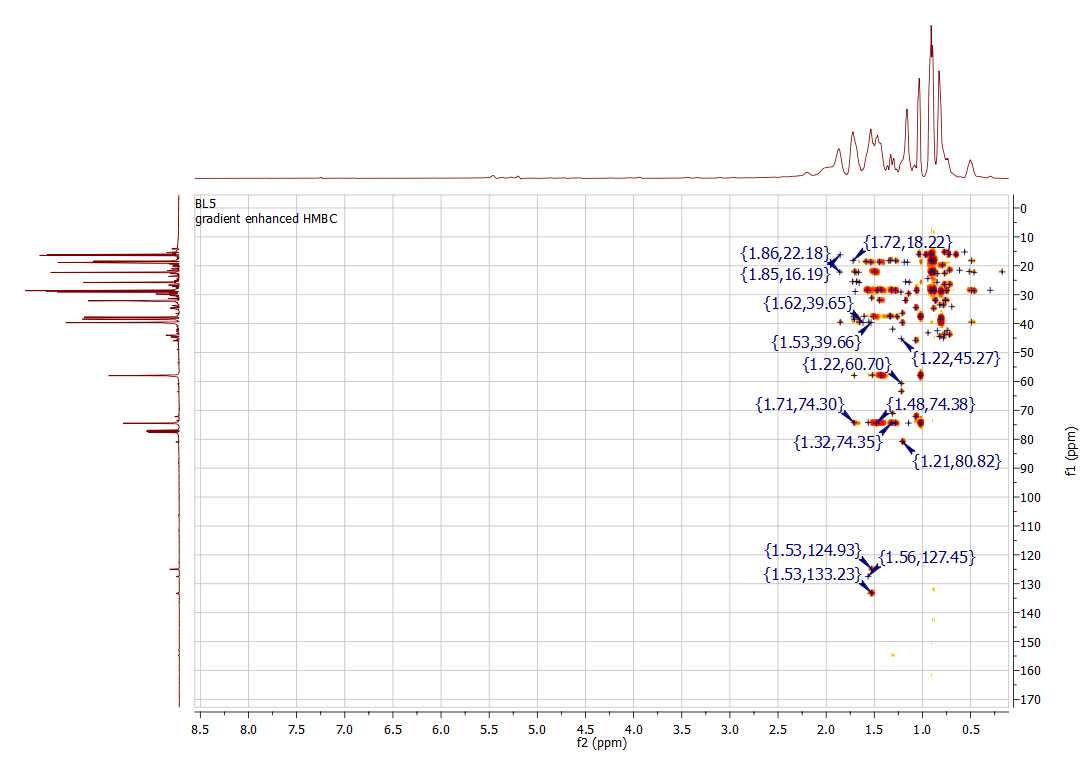
**

**Fig S7**: **HMBC spectrum of compound 21.**


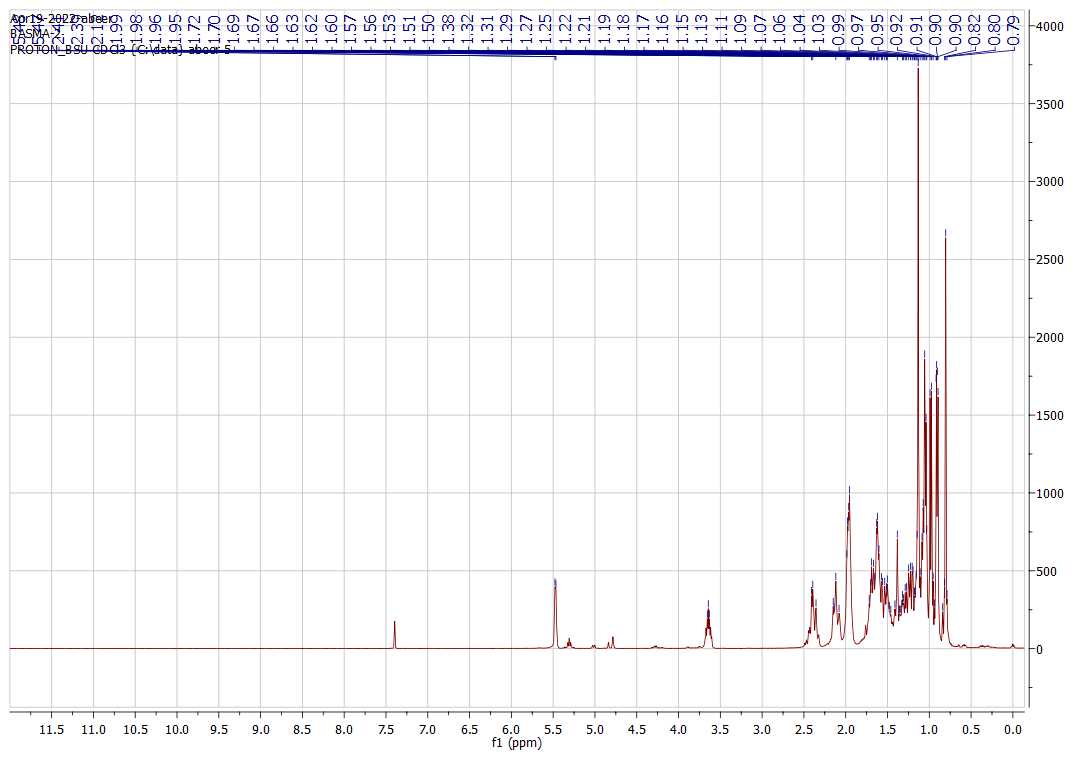


**Fig S8**: **^1^H- NMR spectrum of compound 22 (400MHz, CDCl_3_).**

**
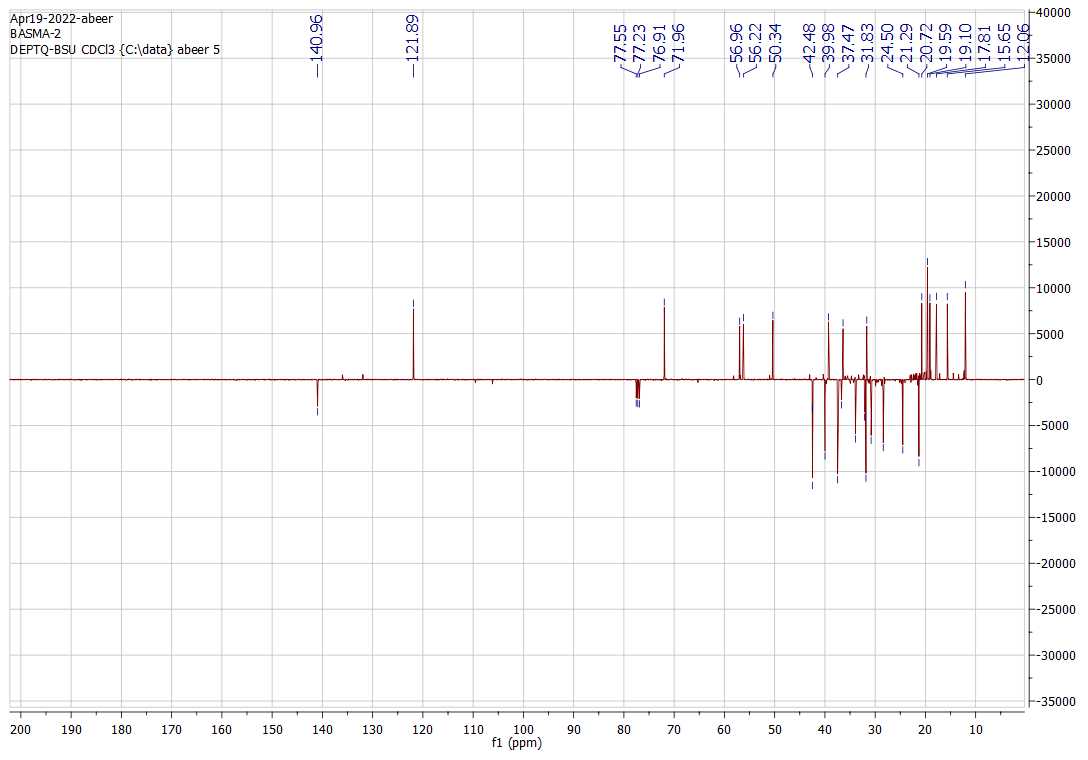
**

**Fig S9**: **DEPT-Q spectrum of compound 22 (100MHz, CDCl_3_).**

**
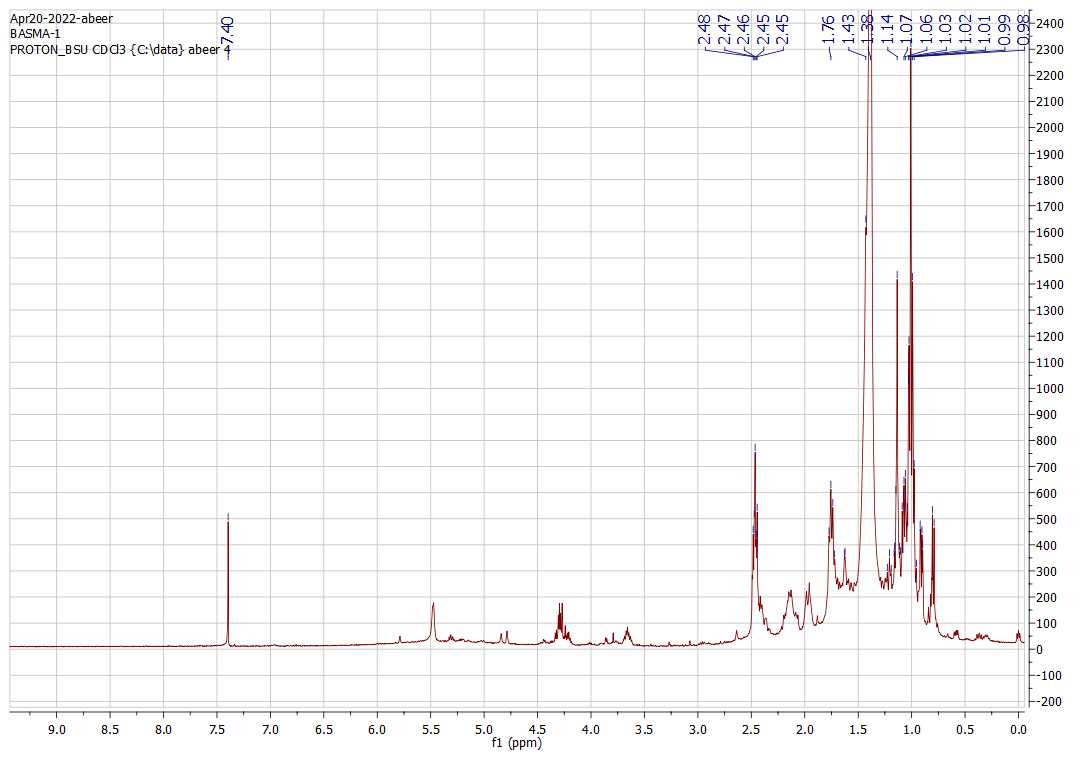
**

**Fig S10**: **^1^H- NMR spectrum of compound 23 (400MHz, CDCl_3_).**

**
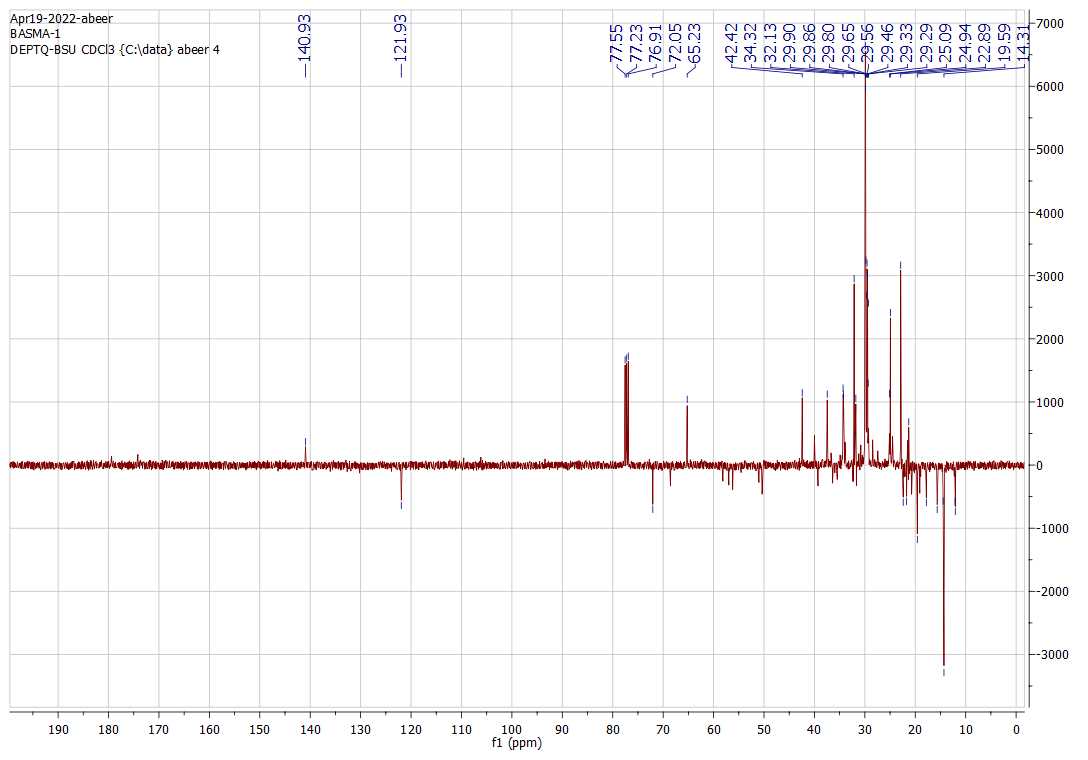
**

**Fig S11**: **DEPT-Q spectrum of compound 23 (100MHz, CDCl_3_).**

**
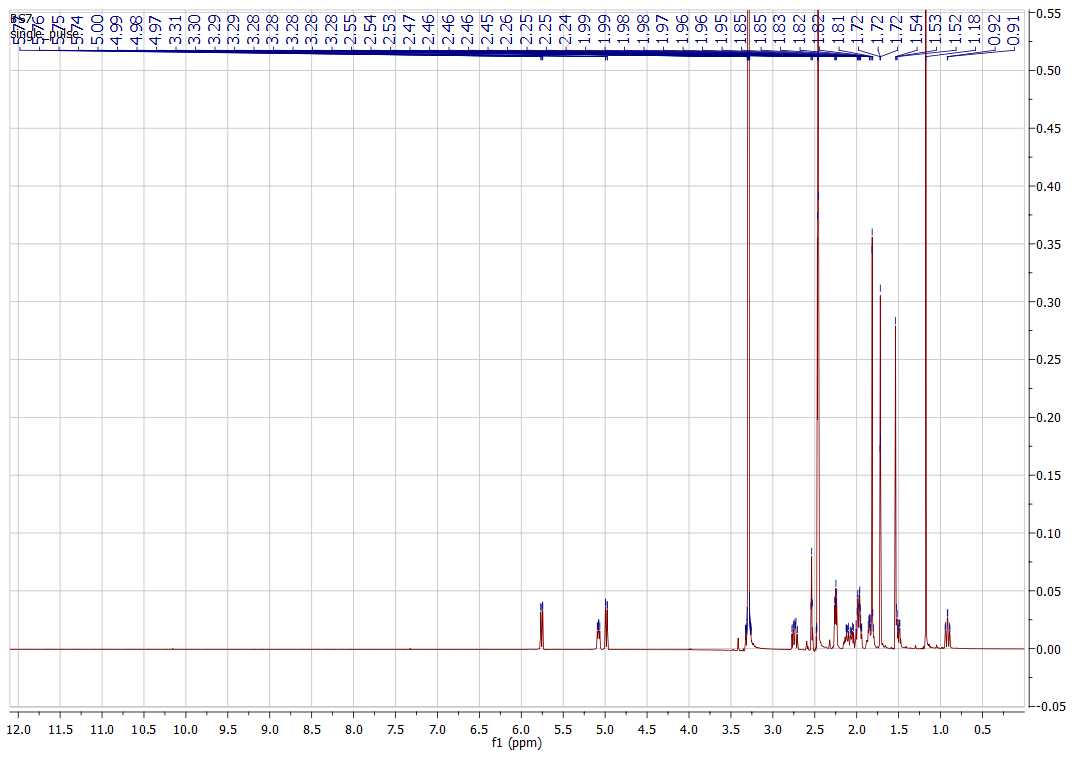
**

**Fig S12**: **^1^H- NMR spectrum of compound 24 (500MHz, DMSO-*d*_6_).**

**
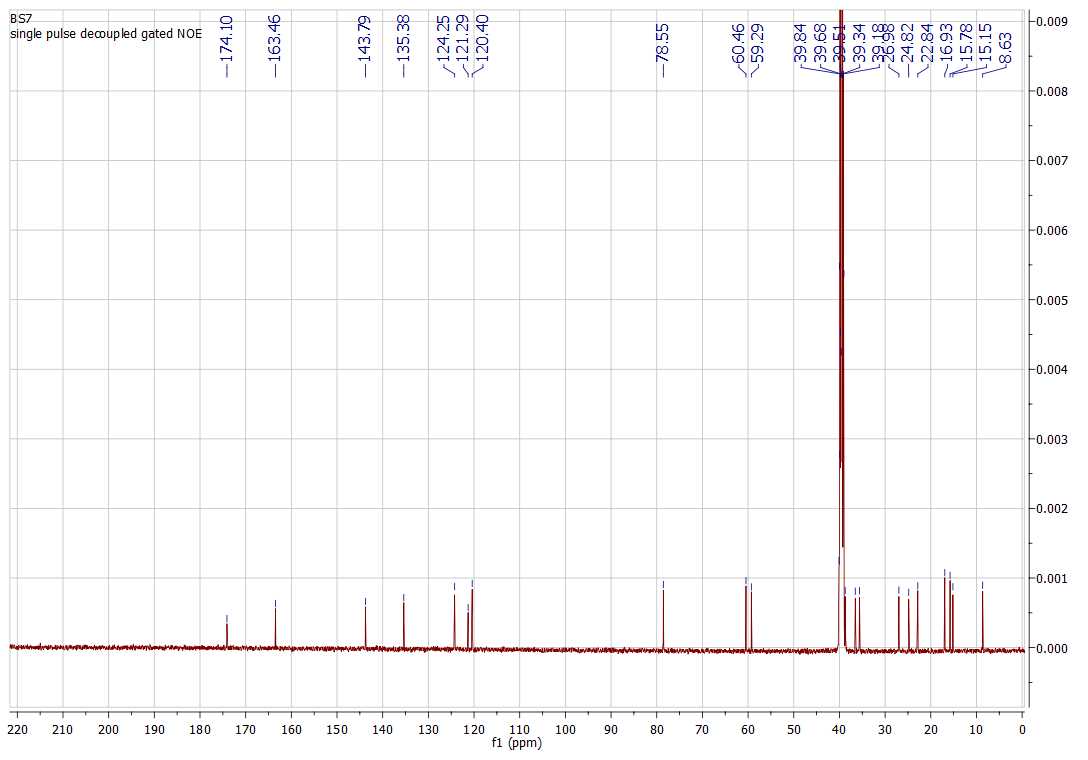
**

**Fig S13**: **^13^C- NMR spectrum of compound 24 (125MHz, DMSO-*d*_6_).**


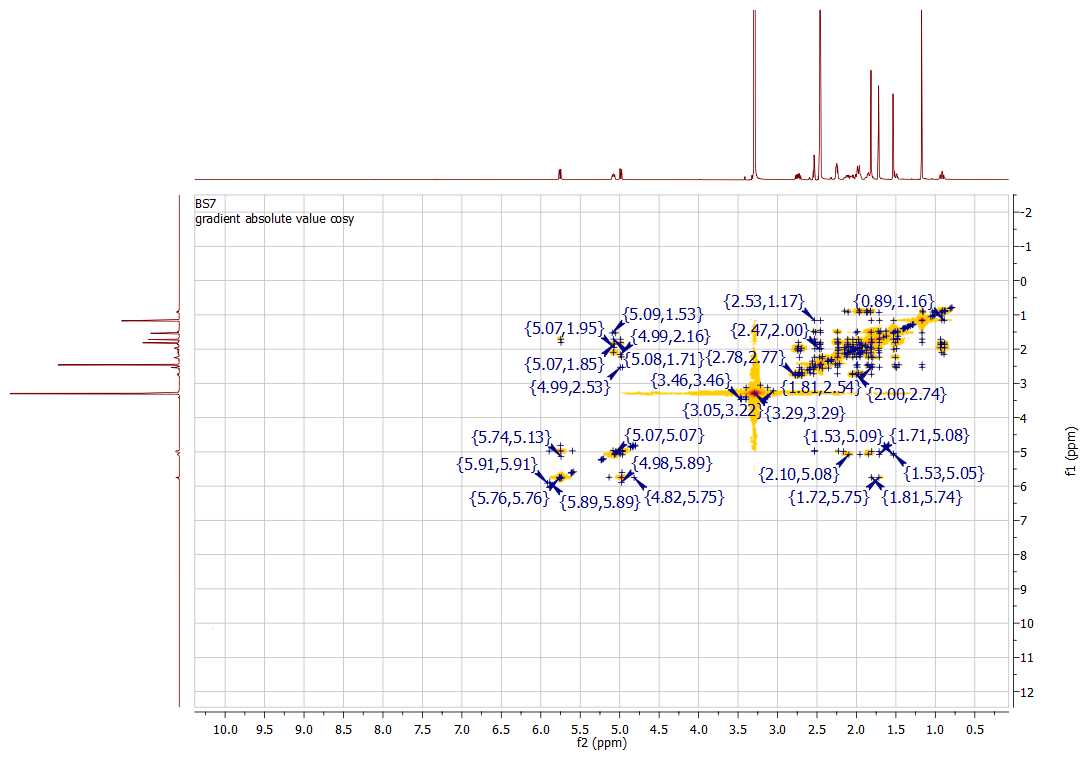


**Fig S14**: **^1^H-^1^H COSY spectrum of compound 24.**

**
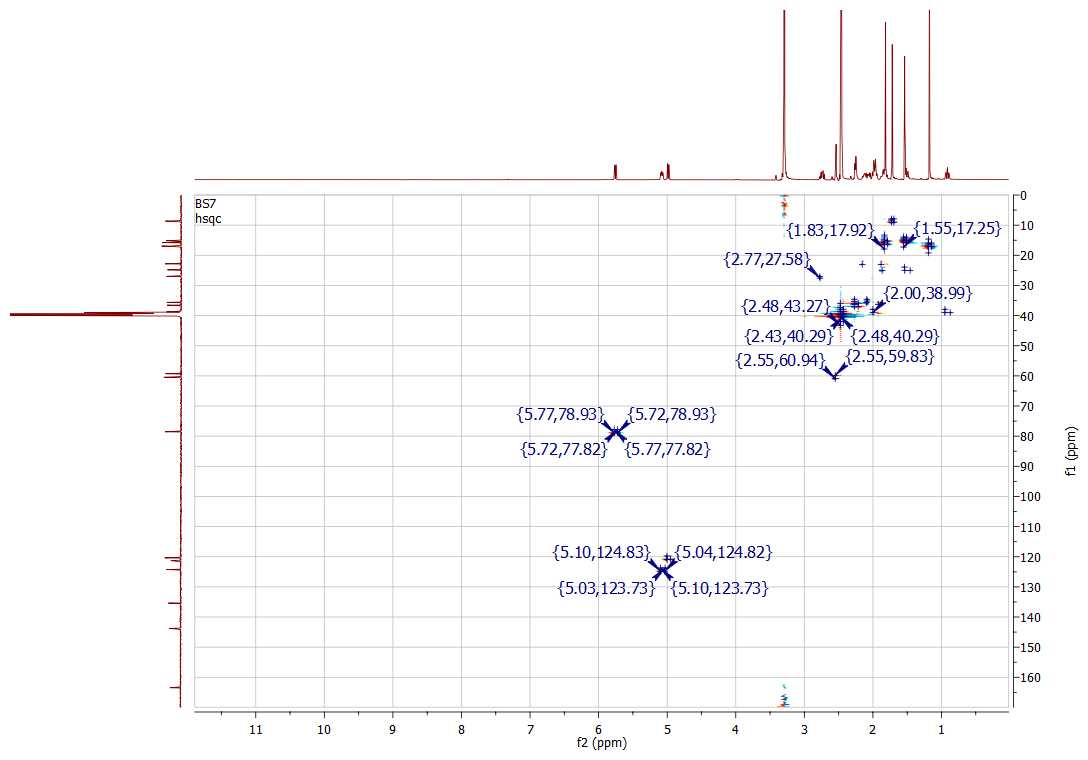
**

**Fig S15**: **HSQC spectrum of compound 24.**

**
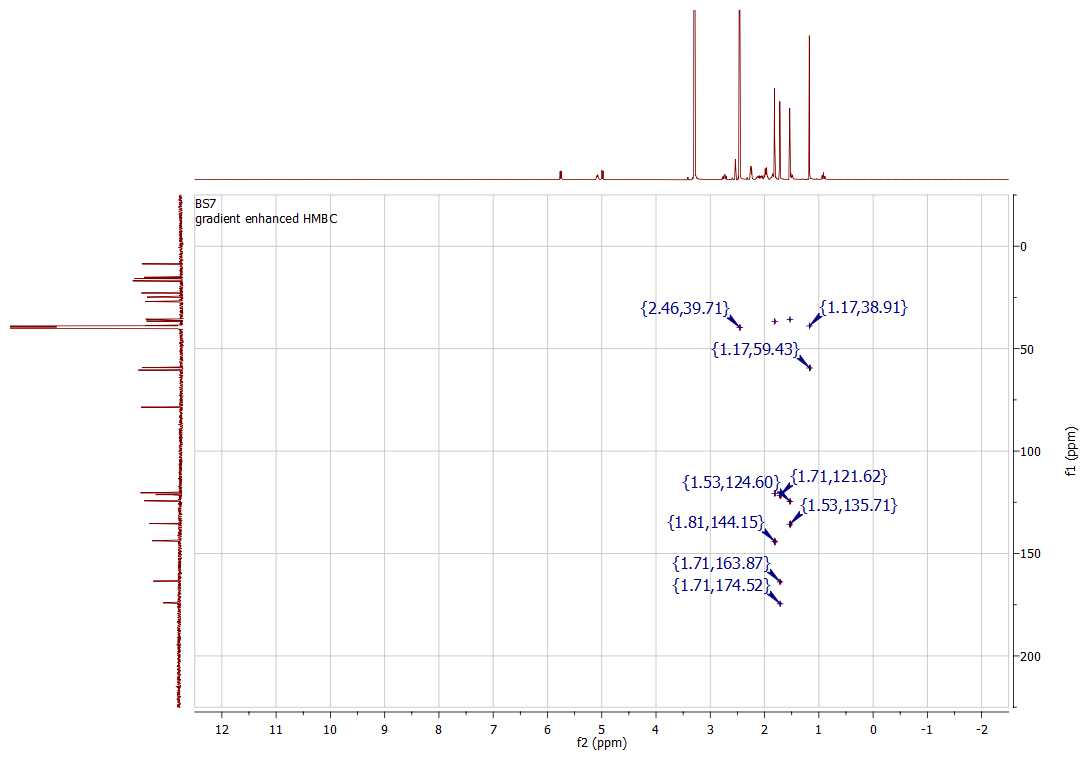
**

**Fig S16**: **HMBC spectrum of compound 24.**


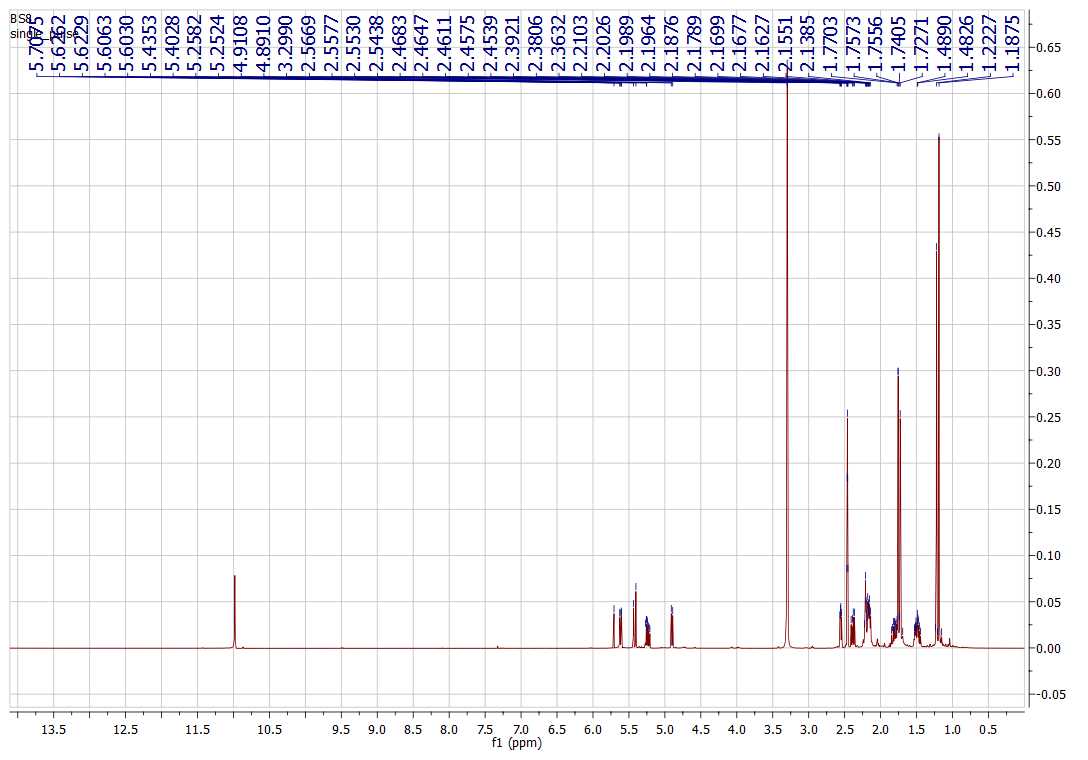


**Fig S17**: **^1^H- NMR spectrum of compound 25 (500MHz, DMSO-*d*_6_).**

**
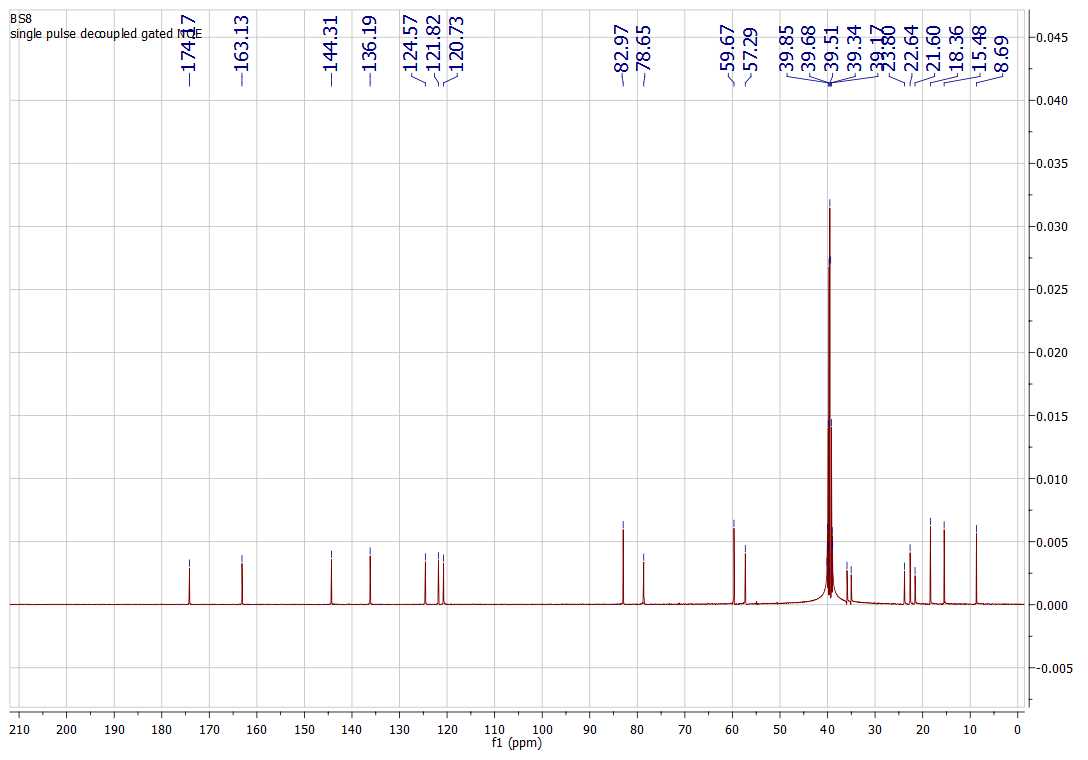
**

**Fig S18**: **^13^C- NMR spectrum of compound 25 (125MHz, DMSO-*d*_6_).**


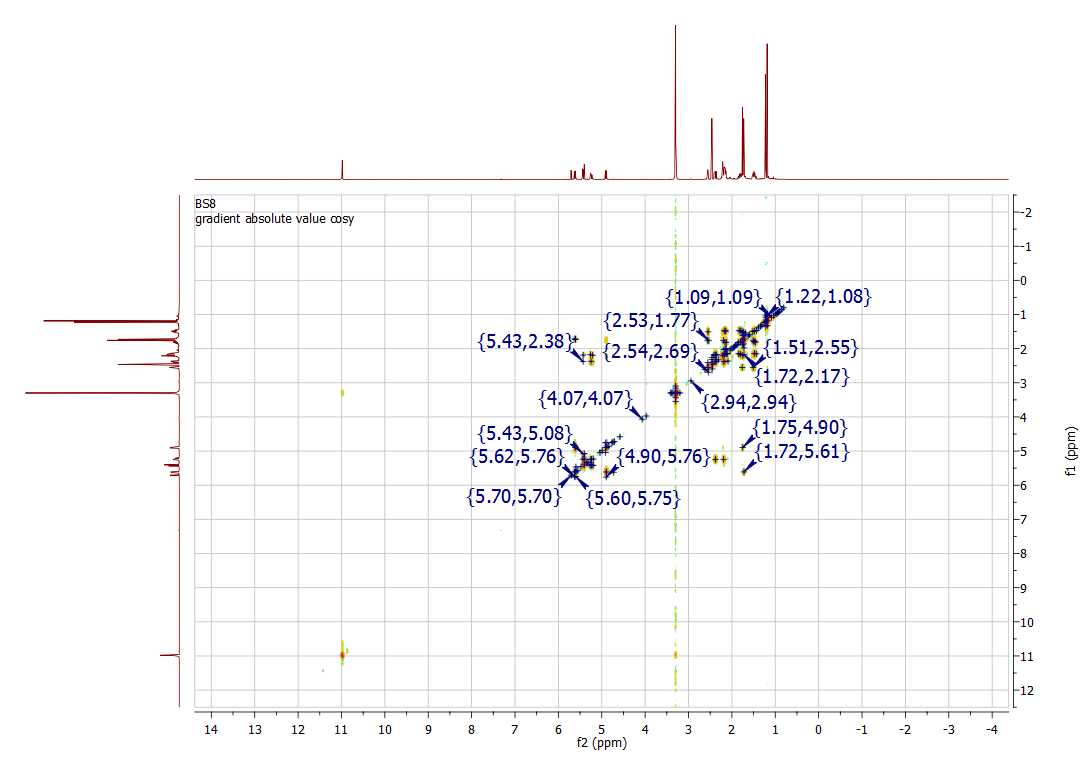


**Fig S19**: **^1^H-^1^H COSY spectrum of compound 25.**

**
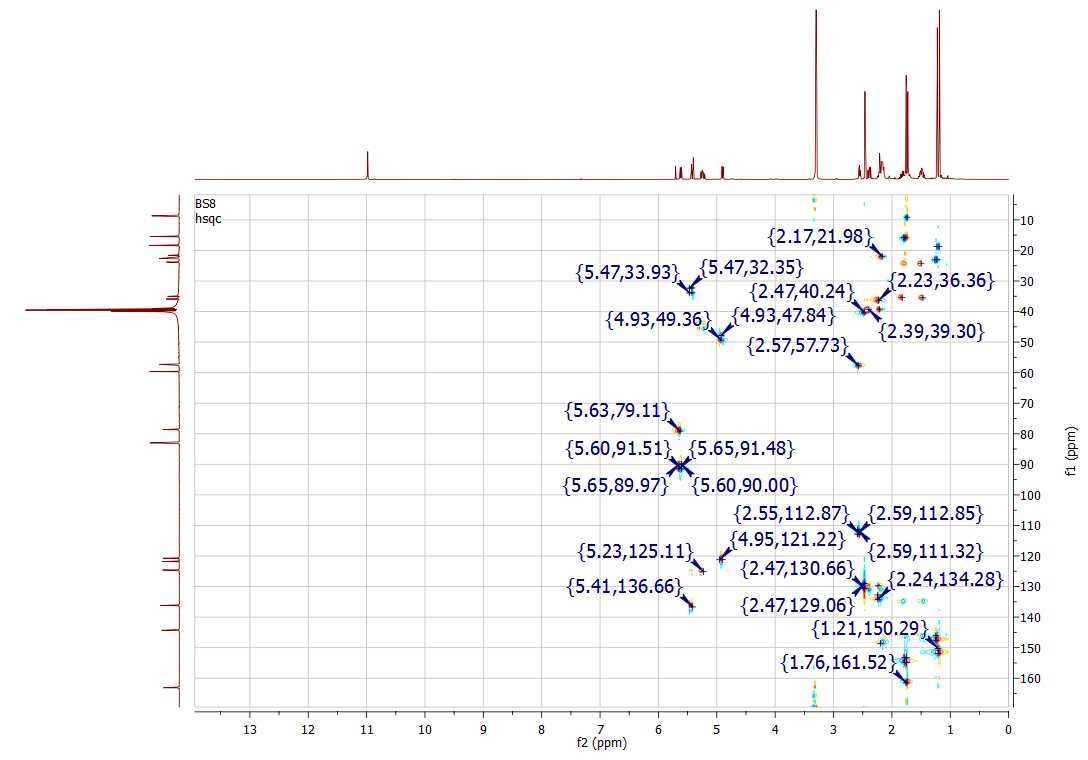
**

**Fig S20**: **HSQC spectrum of compound 25.**

**
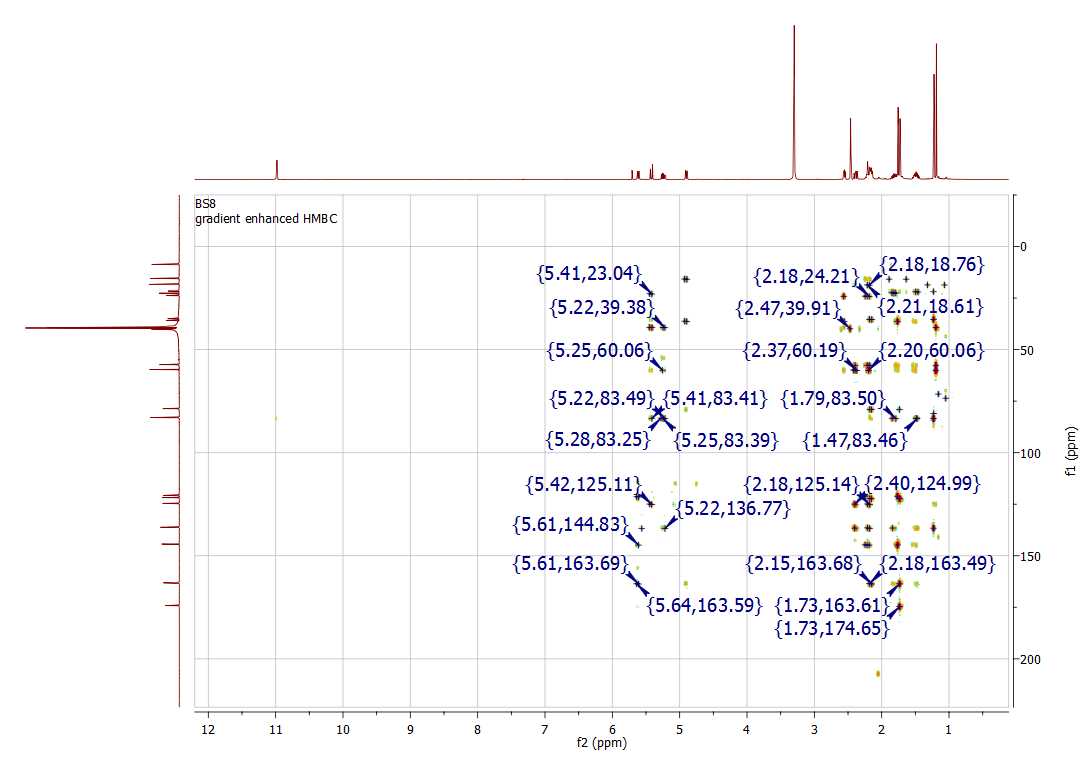
**

**Fig S21**: **HMBC spectrum of compound 25.**


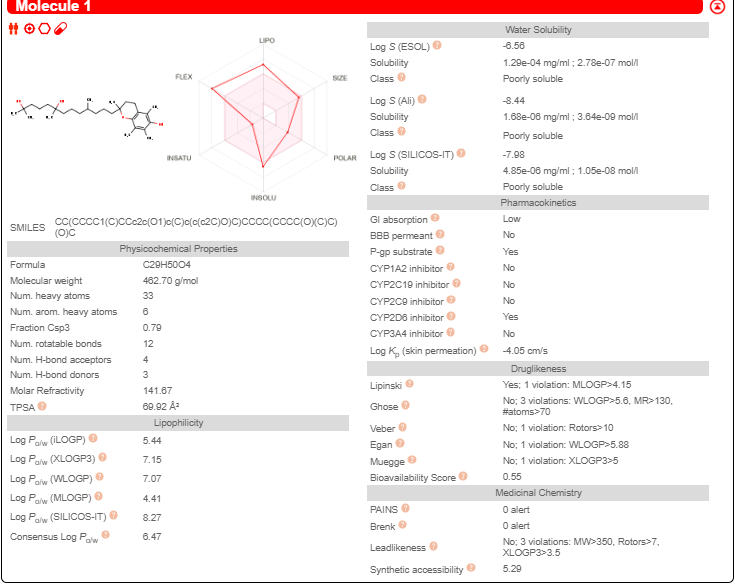


**Fig S22 : SwissADME analysis of compound 17.**
